# Investigating the feasibility of machine learning to guide personalized red blood cell (RBC) transfusion: analyzing the heterogeneity of RBC transfusion in septic patients with hemoglobin levels of 7–9 g/dL based on the causal forest model

**DOI:** 10.3389/fphar.2025.1615618

**Published:** 2025-08-28

**Authors:** Penglei Yang, Jun Yuan, Jie He, Lina Yu, Xue Gu, Xizhen Ding, Qihong Chen

**Affiliations:** ^1^ Department of Critical Care Medicine, Jiangdu People’s Hospital Affiliated to Yangzhou University, Yangzhou, China; ^2^ Department of Emergency Medicine, Yangzhou Jiangdu Traditional Chinese Medicine Hospital, Yangzhou, China

**Keywords:** sepsis, causal forest, red blood cell transfusion, heterogeneity analysis, personalized treatment

## Abstract

**Background:**

This study utilized the causal forest algorithm to explore the heterogeneity of treatment effects of low-dose red blood cell (RBC) transfusion on the 90-day survival rate of sepsis patients with hemoglobin (Hb) levels of 7–9 g/dL to develop personalized transfusion strategies.

**Methods:**

The data of patients the met the Sepsis-3 criteria with a minimum Hb level of 7–9 g/dL were obtained from the MIMIC-IV and MIMIC-III databases and divided into RBC transfusion and non-transfusion groups. Patients in both groups were paired using a propensity score matching analysis (PSM) after which a causal forest model was constructed using MIMIC-IV data. The model’s accuracy was analyzed using out-of-bag data. Individual treatment effects (ITE) of MIMIC-III patients were predicted and categorized into four subgroups: Quantile1 to Quantile4, based on the effect size. Kaplan-Meier survival curves were established for each Quantile to determine the survival rates.

**Results:**

The MIMIC-IV and MIMIC-III database comprised 1,652 and 868 patients, with 826 (50%) and 434 (50%) in the RBC transfusion group, respectively, after PSM. The mean prediction coefficient estimated by the causal forest was 1.00 with a standard error of 0.57, while the differential forest prediction coefficient was 1.64 with a standard error of 0.48, demonstrating the model’s ability to effectively identify differences in the impact of transfusion on survival rates among individuals. There was significant heterogeneity in the ITE among patients in the MIMIC-III validation cohort. Moreover, the ITE values were divided into Quantile1: −5.4% (−8.0%, −3.9%), Quantile2: −2.1% (−2.6%, −1.7%), Quantile3: −0.5% (−0.1%, +0.1%), and Quantile 4: +3.6% (+2.0%, +6.6%). The Kaplan-Meier curves and the log-rank test demonstrated that the RBC transfusion decreased the survival of patients in Quantile1 (p < 0.001) and Quantile2 (p = 0.011) but increased the survival of patients in Quantile4 (p < 0.001).

**Conclusion:**

RBC transfusions among sepsis patients with Hb levels of 7–9 g/dL exhibit heterogenous treatment effects, which reduces the mortality of patients with high ITE. Although the causal forest model can guide personalized transfusion in these cases, randomized controlled trials are needed to validate these findings.

## Graphical Abstract

**Figure:**
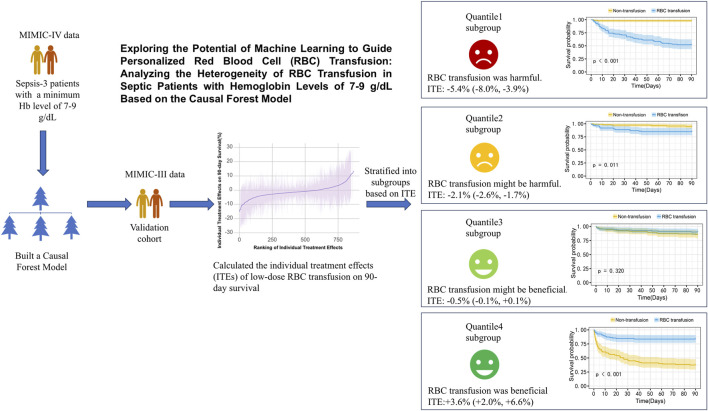
Exploring the Potential of Machine Learning to Guide Personalized Red Blood Cell (RBC) Transfusion: Analyzing the Heterogeneity of RBC Transfusion in Septic Patients with Hemoglobin Levels of 7–9 g/dL.

## Introduction

Sepsis is a life-threatening organ dysfunction caused by a dysregulated immune response to infection (Evans, 2018) and is associated with high incidence and mortality rates. Approximately 19 million global cases of sepsis are reported annually, resulting in five million deaths and the World Health Organization has thus prioritized the diagnosis and treatment of sepsis globally (World Health Organization, 2017). Blood transfusion is an important additional therapy for patients with sepsis (Evans et al., 2021). Treating sepsis requires enhancing oxygen delivery and reducing tissue hypoxia. Hemoglobin (Hb) plays a vital role in facilitating oxygen delivery. Sepsis patients are particularly susceptible to anemia because of hemolysis, hemorrhage, fluid resuscitation, inflammatory responses, and underlying conditions (Qi and Peng, 2021). Yang et al. reported that elderly septic patients with hemoglobin levels below 10 g/dL had a significantly increased risk of mortality (Yang et al., 2023). Studies suggest transfusion below 7 g/dL reduces mortality risk. However, its effects on sepsis patients with Hb levels of 7–9 g/dL remain controversial (Hébert et al., 1999; Parsons et al., 2011; Perner et al., 2012; Holst et al., 2014; Rosland et al., 2014; Evans et al., 2021).

The recommendation to have a restrictive transfusion strategy for sepsis patients when their Hb levels fall below 7 g/dL has limited evidence (Hébert et al., 1999; Holst et al., 2014; Evans et al., 2021). The TRISS trial (Holst et al., 2014) found no significant difference in mortality rates between using 7 g/dL and 9 g/dL as red blood cell transfusion thresholds. These studies suggested that transfusion may not benefit patients with Hb levels of 7–9 g/dL. However, an RBC transfusion threshold of 9 g/dL was found to reduce mortality in cancer patients with sepsis in the TRICOP trial (Bergamin et al., 2017). In a recent retrospective analysis, transfusion of red blood cells (RBC) at an average Hb level of 8.50 g/dL reduced mortality in sepsis patients with chronic kidney disease (Chen et al., 2024). However, there is potential heterogeneity in the treatment effects of transfusions for sepsis patients with Hb levels of 7–9 g/dL. Adopting a uniform transfusion threshold of Hb 7 g/dL is thus not suitable for all sepsis patients. It is thus crucial to identify the factors that influence the effectiveness of RBC transfusions in sepsis patients with Hb levels of 7–9 g/dL for effective transfusion management.

The causal forest model, developed by Susan Athey and Stefan Wager, is a machine-learning method used to estimate causal effects. It was designed to generate many diverse causal trees through subsampling. The model estimates individual treatment effects (ITEs) and provides recommendations for personalized treatment strategies by averaging the predictions of these causal trees (Athey and Imbens, 2016; Wager and Athey, 2018; Inoue et al., 2023). Osawa et al. identified that the causal forest model could identify patients who benefit from polymyxin B hemoperfusion therapy through retrospective data from the JSEPTIC-DIC study (Osawa et al., 2023). Elsewhere, Inoue et al. reported that the causal forest model can guide the administration of personalized statin therapy, potentially reducing cardiovascular disease risk in patients through applied propensity score matching using observational study data (Inoue et al., 2023). However, the ability of the causal forest model to identify sepsis patients with Hb levels between 7–9 g/dL who would benefit from red blood cell transfusions lacks sufficient evidence.

In this study, a causal forest model was constructed based on data of septic patients with Hb levels between 7–9 g/dL from the Medical Information Mart for Intensive Care (MIMIC) -IV database. The model was validated using data from the MIMIC-III database.

## Methods

### Data source

Data were extracted from the MIMIC-IV and MIMIC-III databases maintained by the Massachusetts Institute of Technology. The data was extracted using Navicat and Structured Query Language (SQL) and primarily originated from the intensive care unit of Beth Israel Deaconess Medical Center. Further data processing was conducted through R Studio using R version 4.4.2. The version of RStudio is 2024.12.1 (Build 563). The researchers had permission (License Number: 36463743) to use the data in the MIMIC databases, which are open-access, and adhered to the data use agreement.

### Patient selection

Sepsis patients with a minimum Hb level of 7–9 g/dL admitted to the intensive care unit (ICU) for the first time were enrolled in the study. Sepsis was defined as an infection with a Sequential Organ Failure Assessment (SOFA) score increase of 2 or more from baseline (Levy et al., 2018). The sepsis patient extraction code published by the official MIMIC-IV database team on GitHub (MIT-LCP, 2020) and similar to the infection extraction method by Seymour et al. (2016) and Hu et al. (2023) was employed. According to Nilsson et al., patients who receive ≤670 mL of red blood cells within the first 5 days of ICU admission are deemed to have received low-dose RBC transfusions (Nilsson et al., 2020). Herein, patients with missing Hb data, as well as those with trauma, acute myocardial infarction, gastrointestinal bleeding, and those who received >670 mL of RBC transfusions, were excluded from the study because of the significant differences in the pathological characteristics of sepsis patients with such complications (Perner et al., 2012; Dupuis et al., 2017; Nilsson et al., 2020). The detailed patient selection process is outlined in the Supplementary Material (Supplementary Appendix SA, SB).

### Data extraction

The characteristics of sepsis patients from the MIMIC-IV and MIMIC-III databases, including baseline information, such as age, gender, height, weight, Charlson Comorbidity Index, chronic obstructive pulmonary disease (COPD), chronic heart failure (CHF), and chronic kidney disease (CKD) were extracted. Additional parameters were also collected within 24 h of ICU admission. They included pH (Pondus Hydrogenii), arterial oxygen pressure (PaO_2_), arterial carbon dioxide tension (PaCO_2_), peripheral capillary oxygen saturation (SpO_2_), heart rate (HR), respiratory rate (RR), mean arterial pressure (MAP), white blood cell count (WBC), blood urea nitrogen (BUN), creatinine (Cr), prothrombin time (PT), activated partial thromboplastin time (APTT), lactate (Lac), blood sodium (Na), blood potassium (K), blood glucose (G), body temperature (T), Simplified Acute Physiology Score (SAPS)-II, SAPS-III, Systemic Inflammatory Response Syndrome (SIRS), and Sequential Organ Failure Assessment (SOFA) score among other parameters. The patients’ Hb levels from the first to the fifth day after ICU admission were also recorded. The specific indicators are detailed in Supplementary Appendix SC.

### Statistical analysis

Statistical analyses were conducted using RStudio in R version 4.4.2. Multiple imputations for data with less than 40% missing values in the MIMIC-IV database were done (Hu et al., 2023). The “mice” (v3.18.0) package in R to imputation the missing variables. Detailed imputation methods are outlined in the Supplementary Material (Supplementary Appendix SD). Sepsis patients who received low-dose RBC transfusions were referred to as the RBC transfusion group, while those who did not were the non-transfusion group. Normally distributed continuous variables are presented as means ± standard deviation (X ± s) and were compared using the student's *t*-test to determine if there were any significant differences. Non-normally distributed continuous variables are presented as median (interquartile range) [M(QL, QU)] and were compared using the Mann-Whitney U test to determine whether the distributions were significantly different. Categorical variables are expressed as rates, and their distribution was compared using the χ^2^ chi-square test. The statistical significance level was set at *P* < 0.05.

Incorporating all variables into the model would increase the risk of overfitting because of the many variables included. As such, the Lasso regression and the Boruta model were used to select variables associated with 90-day mortality as covariates for propensity score matching (PSM) analysis by the “glmnet” (v4.1-9) package, the “Boruta” package (v8.0.0), and the “MatchIt” (v4.7.2) package. The PSM analysis was conducted using the intersection of variables selected by Lasso regression and the Boruta algorithm. PSM was performed using the nearest neighbor matching method without replacement. The matching ratio between the RBC transfusion and non-transfusion groups was 1:1, while the caliper value was 0.10.

The “lcmm” package (v2.2.1) was used to perform a latent class mixed models on Hb levels during the first 3 days after ICU admission. The patients were assigned to the latent classes (trajectory groups) based on their maximum posterior probability. Each class represents a different longitudinal pattern characterized by the estimated trajectory parameters. The Hb trajectory was included as a covariate in the casual forest model.

**FIGURE 1:**
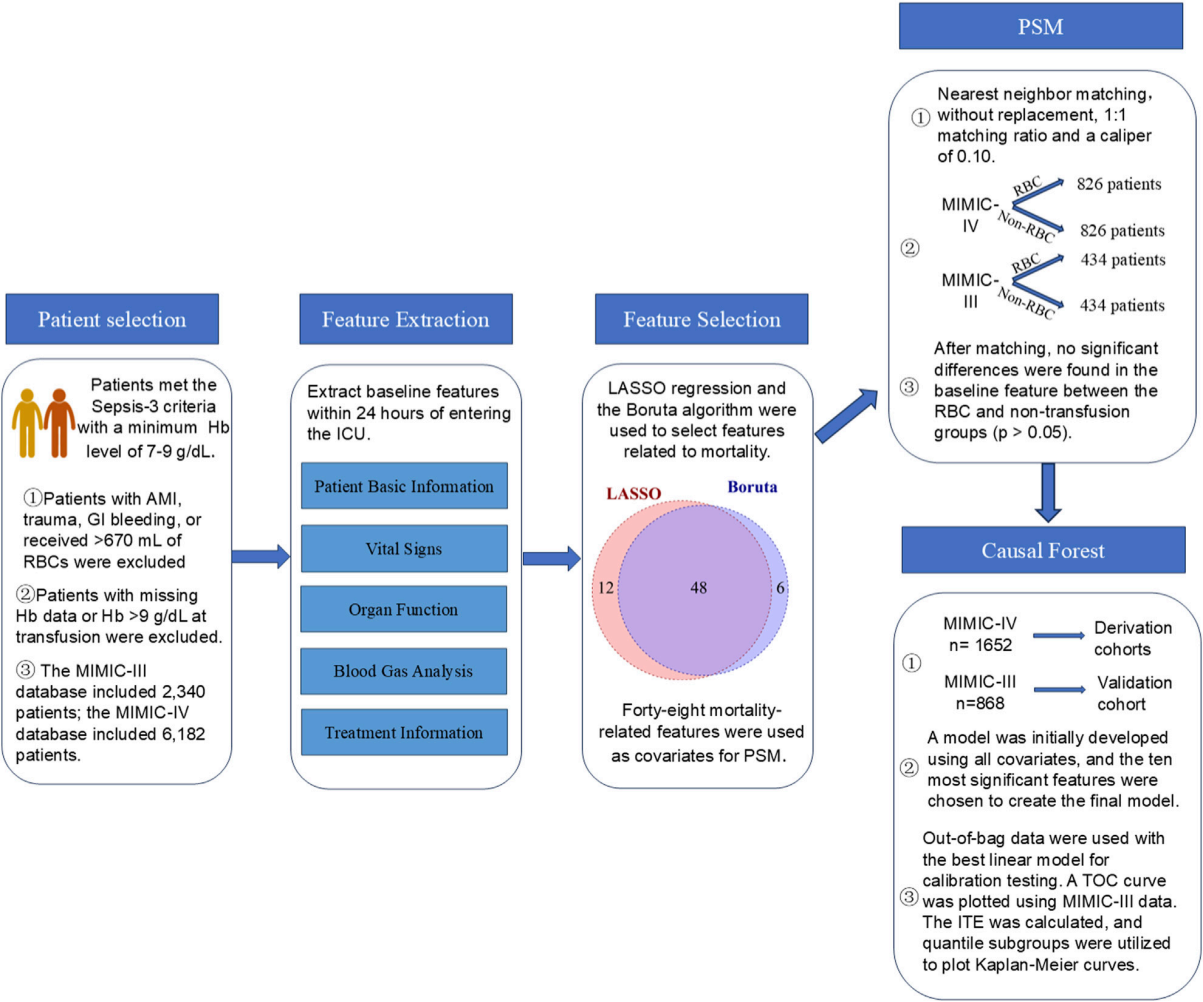
A flow chart of the statistical analysis process.

MIMIC-IV data was used as the derivation cohort, while MIMIC-III data was used as the validation cohort. The “grf” (v2.40) package in R was employed to construct a causal forest model to predict the treatment effect of RBC transfusion on 90-day survival. An ensemble of 5,000 causal trees was erected using the honest splitting method to minimize model overfitting. In this approach, each tree algorithm used a randomly selected 50% subsample from the training set (without replacement) to build the tree structure. The subsample was then split in half. The first half was used to construct the tree structure, while the second half was used for prediction. The remaining hyperparameters were screened using an automatic hyperparameter optimization system. The model was calibrated by fitting the best linear fit of the regression of the observed association on the predicted association (Inoue et al., 2023). The model’s features were displayed using bar charts. Currently, there is no standardized method for variable selection in causal forest models. We tested different numbers of top important features, ranging from the top five to the top twenty, as well as incorporating all available variables. We found that both too many or too few variables reduced predictive performance and ability to detect treatment heterogeneity. Ultimately, we selected top ten ranked variables as covariates in the final model. The final model was reconstructed using the top ten ranked features based on feature importance, and its optimal linear function accuracy was assessed using out-of-bag data. The individual treatment effect (ITE) for patients in the MIMIC-III validation set was predicted, followed by plotting the total operating characteristic (TOC) curve. The Area Under the TOC Curve (AUTOC) was subsequently calculated to evaluate the model’s discriminative ability. Patients from the MIMIC-IV training set and the MIMIC-III validation were classified into four subgroups, Quantile1 to Quantile4, based on ITE values, with ITE values increasing from Quantile1 to Quantile4. Kaplan-Meier survival curves for 90-day survival were plotted to evaluate the survival differences between the 4 subgroups, followed by a log-rank test to compare the discrepancies. Figure 1 is a flow chart of the statistical analysis. Supplementary Appendix SE shows R code for statistical analysis.

## Results

### Baseline characteristics of sepsis patients

**FIGURE 2:**
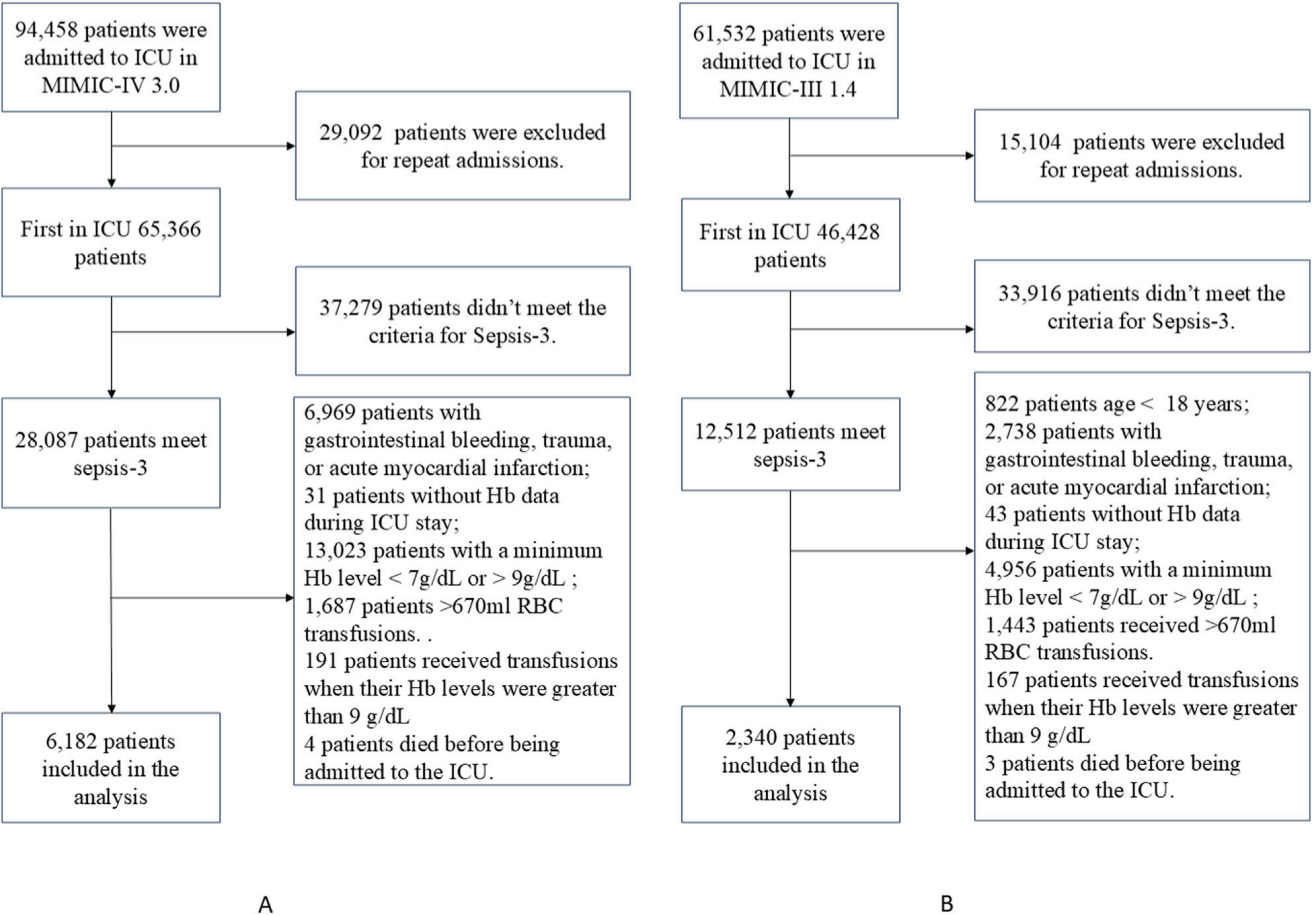
Flowcharts for patient selection from the MIMIC-IV dataset **(A)** and the MIMIC-III dataset **(B)**.

A total of 28,087 and 12,512 patients met the Sepsis-3 criteria in the MIMIC-IV and MIMIC-III databases, respectively. After applying the exclusion criteria, 6,182 patients in the MIMIC-IV and 2,340 patients in the MIMIC-III databases were included, with 857 (13.86%) and 458 (19.57%) belonging to the RBC transfusion group. Figure 2 illustrates the patient screening process. The baseline characteristics of the two groups of patients are detailed in Supplementary Table S1.

### Feature selection and propensity score matching analysis

The LASSO regression and Boruta model were used to select variables associated with 90-day mortality as covariates for PSM analysis. LASSO regression and Boruta model identified 60 and 54 variables, respectively, associated with mortality. A combination of LASSO regression and Boruta model results led to the selection of 48 variables. The specific process of variable selection is detailed in Supplementary Appendix SE.

PSM analysis included 48 features as covariates. There were 1,652 matched patients in the MIMIC-IV database: 826 in the RBC transfusion group and 826 in the non-transfusion group. In contrast, the MIMIC-III database had 868 matched patients: 434 in the RBC transfusion group and 434 in the non-transfusion group. In the MIMIC-IV database, the 24-h fluid intake was significantly higher in the transfusion group compared to the non-transfusion group (p < 0.001). Notably, after matching, no statistically significant differences were observed in the remaining baseline characteristics between the RBC transfusion and non-transfusion groups in either database (p > 0.05) (Table 1).

**TABLE 1:** Baseline characteristics of patients in the RBC transfusion and non-transfusion groups after propensity score matching.

Variable	MIMIC-IV			MIMIC-III		
	Non-transfusion *N = 5,325*	RBC transfusion *N = 857*	p	Non-transfusion *N = 5,325*	RBC transfusion *N = 857*	p
Male (%)	406 (49.15%)	378 (45.76%)	0.183	215 (49.54%)	199 (45.85%)	0.308
Age (years)	77.58 [66.50; 90.78]	76.73 [65.13; 91.00]	0.449	75.00 [64.65; 88.50]	75.45 [63.93; 88.00]	0.928
Weight (Kg)	100.00 [90.00; 112.00]	101.00 [89.00; 115.00]	0.389	104.00 [92.00; 116.00]	104.00 [91.00; 118.00]	0.793
HR_max (bpm)	83.91 [75.57; 93.95]	83.57 [75.96; 95.10]	0.508	87.31 [79.81; 96.93]	87.18 [79.08; 97.36]	0.973
MBP_min (mmHg)	56.00 [50.00; 61.00]	56.00 [49.00; 61.00]	0.379	55.00 [49.00; 61.00]	55.17 [49.00; 60.75]	0.992
MBP_mean (mmHg)	72.69 [68.44; 77.52]	72.62 [68.94; 77.60]	0.663	73.50 [68.56; 77.76]	72.59 [67.50; 78.43]	0.643
RR_max	26.00 [23.00; 30.00]	26.00 [23.00; 30.00]	0.693	27.00 [24.00; 31.00]	26.00 [23.00; 30.00]	0.407
RR_mim	17.79 [15.88; 20.30]	17.97 [16.04; 20.35]	0.399	18.21 [16.10; 20.93]	18.04 [15.67; 20.91]	0.399
T_min (°C)	36.33 [35.67; 36.61]	36.33 [35.68; 36.56]	0.698	35.90 [35.41; 36.39]	35.83 [35.50; 36.44]	0.811
T_max (°C)	37.28 [37.00; 37.80]	37.33 [37.00; 37.83]	0.298	37.50 [37.00; 38.00]	37.56 [37.00; 38.04]	0.856
T_mean (°C)	36.76 [36.53; 37.09]	36.79 [36.53; 37.07]	0.575	36.75 [36.43; 37.13]	36.78 [36.40; 37.16]	0.650
SPO_2__min (%)	93.00 [91.00; 95.00]	93.00 [91.00; 95.00]	0.458	93.00 [90.00; 94.00]	93.00 [90.00; 95.00]	0.828
SPO_2__mean (%)	97.74 [96.67; 98.73]	97.74 [96.51; 98.80]	0.901	97.62 [96.34; 98.70]	97.72 [96.43; 98.72]	0.706
G_min (mg/dL)	93.00 [82.00; 115.00]	96.00 [83.00; 117.00]	0.190	90.00 [78.00; 104.75]	91.00 [79.00; 110.00]	0.270
G_max (mg/dL)	170.00 [143.00; 201.75]	170.00 [145.00; 208.00]	0.478	166.50 [138.25; 195.75]	168.50 [140.00; 203.00]	0.444
G_mean (mg/dL)	127.81 [115.00; 147.33]	128.79 [116.67; 149.95]	0.254	124.63 [110.08; 136.49]	124.69 [112.92; 140.66]	0.201
PLT_min (10^9^/L)	135.00 [97.00; 190.00]	131.00 [97.00; 190.75]	0.791	139.00 [105.00; 199.75]	136.00 [100.00; 217.75]	0.940
PLT_max (10^9^/L)	176.50 [133.00; 243.00]	176.00 [131.00; 250.00]	0.908	183.50 [137.00; 254.75]	181.00 [134.25; 260.25]	0.881
WBC_min (10^9^/L)	8.70 [6.10; 12.40]	9.20 [6.40; 12.30]	0.309	8.80 [5.90; 12.18]	8.85 [6.40; 12.50]	0.488
WBC_max (10^9^/L)	13.60 [9.63; 18.30]	13.70 [10.10; 18.275]	0.560	12.75 [9.025; 17.48]	12.85 [9.325; 17.98]	0.678
HCT_min	24.50 [23.00; 26.20]	24.30 [23.00; 26.08]	0.404	23.90 [22.00; 25.80]	23.60 [22.00; 25.78]	0.958
HCT_max	30.30 [27.90; 33.20]	30.20 [27.925; 32.80]	0.668	32.20 [28.60; 36.00]	32.00 [28.50; 35.00]	0.662
HCO_3__min	22.00 [19.00; 24.00]	21.50 [19.00; 24.00]	0.367	22.00 [20.00; 24.00]	22.00 [20.00; 24.00]	0.417
BUN_min (mg/dL)	17.00 [12.00; 30.00]	18.00 [12.00; 29.00]	0.432	17.00 [12.00; 27.00]	18.00 [13.00; 29.00]	0.068
BUN_max (mg/dL)	21.00 [14.00; 35.00]	21.00 [14.00; 35.00]	0.916	19.00 [14.00; 32.00]	21.00 [15.00; 34.750]	0.139
Cr_max (mg/dL)	1.00 [0.725; 1.70]	1.10 [0.80; 1.60]	0.645	1.00 [0.70; 1.70]	1.10 [0.80; 1.60]	0.672
Cr_min (mg/dL)	0.80 [0.60; 1.30]	0.90 [0.60; 1.30]	0.489	0.90 [0.60; 1.40]	0.90 [0.70; 1.30]	0.691
Na_min (mmol/L)	137.00 [134.00; 139.00]	137.00 [134.00; 139.00]	0.612	135.00 [133.00; 137.00]	135.00 [133.00; 137.00]	0.943
Na_max (mmol/L)	139.00 [136.00; 142.00]	139.50 [137.00; 142.00]	0.544	139.00 [137.00; 142.00]	140.00 [138.00; 142.00]	0.644
K_max (mmol/L)	4.50 [4.20; 4.90]	4.50 [4.10; 4.90]	0.532	4.80 [4.20; 5.40]	4.80 [4.20; 5.40]	0.809
INR_max	1.40 [1.30; 1.70]	1.40 [1.30; 1.70]	0.194	1.40 [1.30; 1.60]	1.40 [1.30; 1.70]	0.935
PT_max(s)	16.00 [14.20; 18.40]	15.60 [14.00; 18.38]	0.086	15.60 [14.20; 17.58]	15.65 [14.50; 17.50]	0.771
APTT_max(s)	34.45 [29.90; 44.45]	34.60 [29.93; 43.95]	0.702	36.50 [30.70; 46.03]	36.95 [30.93; 46.28]	0.741
Lac_max(s)	2.40 [1.60; 3.50]	2.40 [1.60; 3.50]	0.709	2.30 [1.60; 3.50]	2.40 [1.60; 3.40]	0.730
PH_min	7.33 [7.28; 7.38]	7.33 [7.28; 7.37]	0.659	7.33 [7.27; 7.39]	7.33 [7.28; 7.38]	0.806
PO_2__min (mmHg)	95.00 [73.00; 122.75]	90.00 [72.00; 121.00]	0.111	87.00 [72.00; 111.00]	87.00 [71.25; 113.75]	0.819
PCO_2__max (mmHg)	45.00 [39.00; 51.00]	45.00 [40.00; 50.00]	0.770	46.00 [40.00; 52.00]	46.00 [40.00; 51.00]	0.726
Charlson	5.00 [4.00; 7.00]	6.00 [4.00; 7.00]	0.392	5.00 [3.00; 7.00]	5.00 [3.00; 6.00]	0.587
SOFA	3.00 [2.00; 5.00]	3.00 [2.00; 5.00]	0.882	5.00 [4.00; 8.00]	5.00 [4.00; 7.75]	0.678
SAPS_II	38.00 [31.00; 49.00]	39.00 [31.00; 49.00]	0.501	37.00 [31.00; 48.00]	38.00 [31.00; 48.00]	0.932
SAPS_III	45.00 [33.00; 66.00]	46.00 [34.00; 65.00]	0.685	43.00 [34.00; 62.00]	45.00 [33.00; 61.00]	0.936
SIRS	3.00 [2.00; 3.00]	3.00 [2.00; 3.00]	0.623	3.00 [2.00; 4.00]	3.00 [2.00; 4.00]	0.845
RDW_min (%)	14.60 [13.40; 16.30]	14.70 [13.60; 16.40]	0.207	14.70 [13.625; 16.38]	14.80 [13.80; 16.50]	0.294
RDW_max (%)	14.90 [13.80; 16.80]	15.20 [14.20; 17.00]	0.061	15.20 [14.00; 16.88]	15.40 [14.40; 17.20]	0.052
ad_Hb	8.80 [8.00; 9.90]	8.90 [8.10; 10.00]	0.194	8.80 [7.90; 9.80]	8.70 [8.20; 9.80]	0.437
VDI (µg/min)	0.05 [0.00; 0.15]	0.06 [0.00; 0.15]	0.872	0.05 [0.00; 0.10]	0.05 [0.00; 0.15]	0.285
Fluid_3h_sum (ml)	114 [0; 857]	161 [0; 873]	0.242	150 [0; 1,002]	164 [0; 1,003]	0.902
Fluid_6h_sum (ml)	1,129 [255; 2,128]	1,049 [387; 2,188]	0.599	1,006 [200; 2072]	1,040 [232; 2,431]	0.860
Fluid_24h_sum (ml)	3,124 [1,594; 4,413]	3,536 [2,173; 5,111]	<0.001	2,632 [1,280; 4,198]	3,065 [958; 4,456]	0.568
Shock (%)	554 (67.07%)	545 (65.98%)	0.677	269 (61.98%)	273 (62.90%)	0.833
Anemia (%)	279 (33.78%)	290 (35.11%)	0.605	114 (26.27%)	125 (28.80%)	0.447
CHF(%)	213 (25.79%)	189 (22.88%)	0.187	108 (24.89%)	131 (30.18%)	0.095
CVD (%)	99 (11.99%)	77 (9.32%)	0.094	34 (7.83%)	29 (6.68%)	0.601
CPD(%)	197 (23.85%)	226 (27.36%)	0.114	102 (23.50%)	104 (23.96%)	0.936
RD (%)	36 (4.36%)	36 (4.36%)	1.000	23 (5.30%)	12 (2.77%)	0.084
CRD (%)	166 (20.10%)	165 (19.98%)	1.000	83 (19.12%)	81 (18.66%)	0.931
Cancer (%)	105 (12.71%)	112 (13.56%)	0.662	77 (17.74%)	74 (17.05%)	0.858
Type of ICU(%)						
MICU	129 (15.62%)	134 (16.22%)	0.856	161 (37.10%)	158 (36.41%)	0.955
M_SICU	136 (16.47%)	147 (17.80%)		-	-	
SICU	176 (21.31%)	169 (20.46%)		70 (16.13%)	73 (16.82%)	
CICU	385 (46.61%)	376 (45.52%)		203 (46.77%)	203 (46.77%)	
Source of infection (%)						
Lung	60 (7.26%)	65 (7.87%)	0.668	45 (10.37%)	45 (10.37%)	0.942
Abdomen	32 (3.87%)	29 (3.51%)		21 (4.83%)	17 (3.92%)	
Urinary	56 (6.78%)	69 (8.35%)		34 (7.83%)	39 (8.99%)	
Blood	153 (18.52%)	139 (16.83%)		70 (16.13%)	69 (15.90%)	
Other	525 (63.56%)	524 (63.44%)		264 (60.83%)	264 (60.83%)	
MV(%)	428 (51.82%)	452 (57.42%)	0.237	295 (67.97%)	296 (68.20%)	0.942

HR, heart rate; MBP, mean blood pressure; RR, respiratory rate; T, temperature; SpO2, oxygen saturation; G, glucose; PLT, platelet; WBC, white blood cell; HCO3, bicarbonate; BUN, blood urea nitrogen; Cr, Creatinine; Na, Sodium; K, potassium; INR, international normalized ratio; PT, prothrombin time; APTT, activated partial thromboplastin time; ALT, alanine aminotransferase; AST, aspartate aminotransferase; Hb, Hemoglobin; Fluid_3h_sum, Volume of intravenous infusion within 3h of ICU, admission; Fluid_6h_sum, Volume of intravenous infusion within 6h of ICU, admission; Fluid_24h_sum, Volume of intravenous infusion within 24h of ICU, admission; AKI, acute kidney injury; VDI_24hmax, Maximum Norepinephrine Equivalent dose within 24 h in ICU; Lac, Lactate; PO2, arterial oxygen partial pressure; PCO_2_, arterial carbon dioxide partial pressure; Charlson, Charlson comorbidity index; SAPS-II, Simplified Acute Physiology Score II; SAPS-III, Simplified Acute Physiology Score III; SIRS, systemic inflammatory response syndrome; SOFA, sequential organ failure assessment; CHF, congestive heart failure; CVD, cerebrovascular disease; CPD, chronic pulmonary disease; RD, rheumatic disease; CRD, chronic renal disease; MV, mechanical ventilation.

The Kaplan-Meier curves and log-rank test in both the MIMIC-IV and MIMIC-III datasets demonstrated no statistically significant differences in the 90-day survival rates between the RBC transfusion and non-transfusion groups after matching (p > 0.05) (Supplementary Figure S1). Similarly, there was no statistically significant difference in Hb levels between the transfusion and non-transfusion groups upon ICU admission in the MIMIC-IV and MIMIC-III datasets (p > 0.05). Hb levels were significantly higher in the transfusion group compared to the non-transfusion group (p < 0.05) on the third, fourth, and fifth days after ICU admission (Supplementary Figure S2; Supplementary Table S1). The trajectories of Hb were classified into three categories after matching: decreasing, stable, and increasing class (Supplementary Figures S3, S4). The relative entropy was 0.759, while the Bayesian Information Criterion (BIC) was 18,688. The Average Posterior Probability of Assignment (APPA) values of the decreasing, stable, and increasing classes were 0.890, 0.904, and 0.808, respectively.

### Causal forest models and survival analysis

**FIGURE 3:**
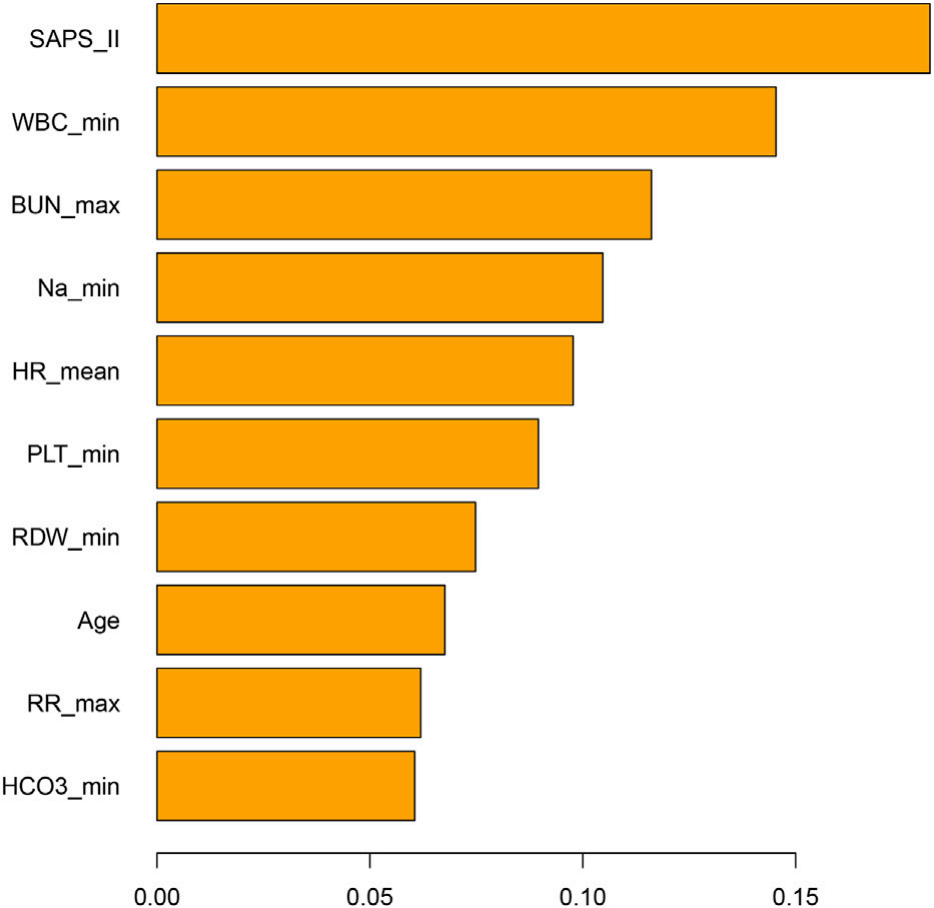
Bar chart illustrating the importance of features in the final causal forest model. The features are the patient’s parameters measured within the first 24 h of admission to the ICU. ICU: Intensive Care Unit; SAPS-II, Simplified Acute Physiology Score II; WBC_min, Minimum white blood cell count within 24 h in ICU; BUN_max, Maximum blood urea nitrogen within 24 h in ICU; Na_min, Minimum sodium within 24 h in ICU; HR_mean, Mean heart rate within 24 h in ICU; PLT_min, Minimum platelet count within 24 h in ICU; RDW_max, Maximum red cell distribution width within the first 24 h of ICU admission; RR_max, Maximum respiratory rate within 24 h in ICU; HCO_3__min, Minimum bicarbonate within 24 h in ICU.

**FIGURE 4:**
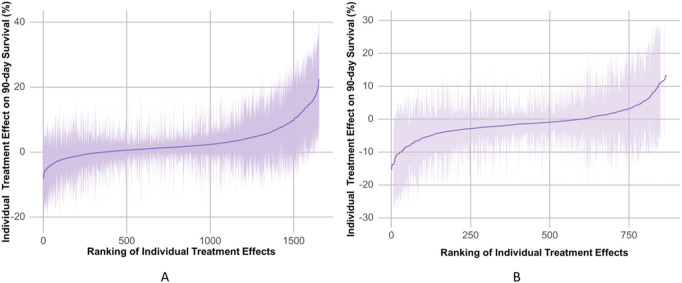
The estimated individual treatment effects (ITEs) distributed in the MIMIC-IV derivation cohorts **(A)** and the MIMIC-III validation cohorts **(B)**. The solid line represents mean of ITEs. The shaded bands represent the 95% confidence interval of ITEs.

**FIGURE 5:**
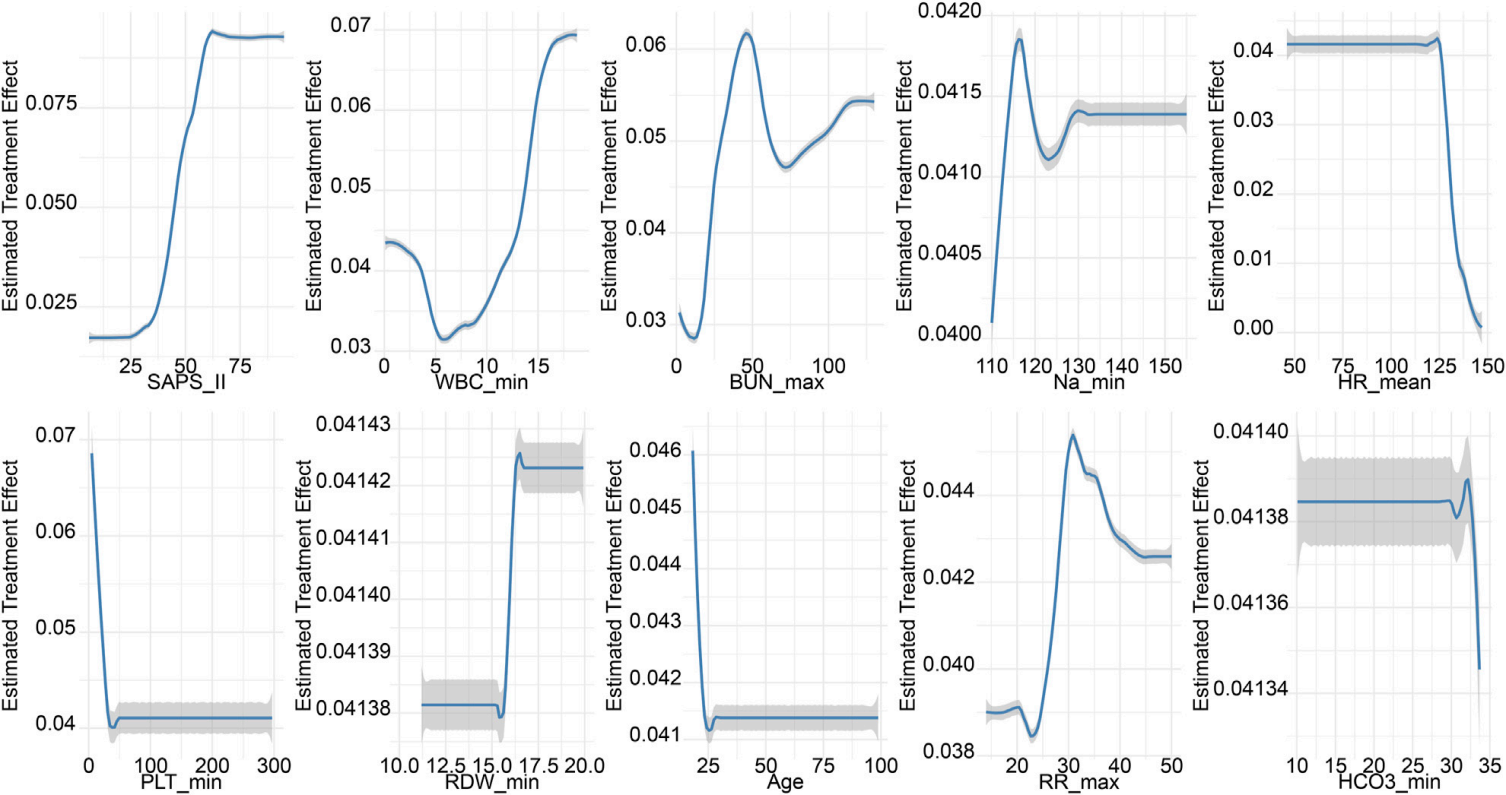
Partial dependence plots (PDPs) for features of the final causal forest model. The PDPs demonstrate how one feature affects the predicted outcome of the final causal forest model averaged across the distribution of the other features.

**FIGURE 6:**
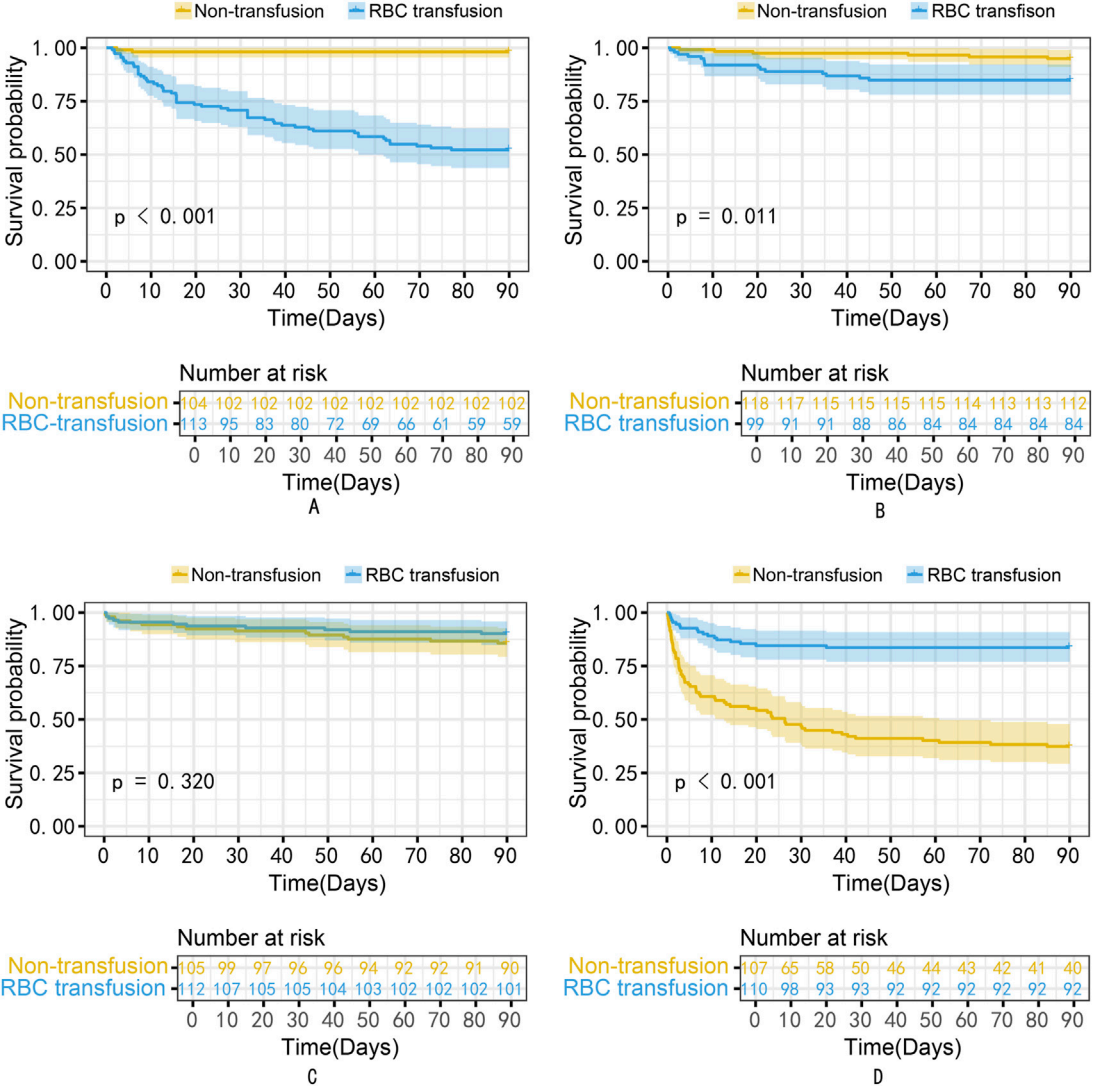
The Kaplan-Meier curves for patients in Quantiles1 to four subgroups of the MIMIC-III validation cohort. **(A)** Quantile1; **(B)** Quantile2; **(C)** Quantile3; **(D)** Quantile4. RBC, Red Blood Cell.

The propensity-matched data from the MIMIC-IV and MIMIC-III databases were the derivation and validation cohort, respectively. All baseline characteristics and Hb trajectories were used as covariates. SAPS-II, minimum WBC (WBC_min), maximum BUN (BUN_max), minimum Na (Na_min), mean HR (HR_mean), minimum PLT (PLT_min), minimum RDW (RDW_min), Age, maximum RR (RR_max), and minimum HCO_3_ (HCO_3__min) within the first 24 h of ICU admission were the top ten features ranked by importance and were used to develop the final model. The importance of each feature in the final model is illustrated in Figure 3. The accuracy of the final causal forest model was validated using the best linear model on out-of-bag data. The mean forest prediction coefficient was 1.00 with a standard error of 0.57, indicating good model accuracy, while the differential forest prediction coefficient was 1.64 with a standard error of 0.48, demonstrating the model’s ability to effectively identify differences in the impact of transfusion on survival rates among individuals. The ranking of individual treatment effects for patients in the MIMIC-IV and MIMIC-III datasets showed heterogeneity in treatment effects among the patients (Figure 4). Noteworthy, the average treatment effect decreased as the treatment proportion increased (Supplementary Figure S5). The area under the Targeted Operating Characteristic curve (AUTOC) was 0.08 ± 0.02. This value indicated that the model effectively identified the treatment effect of RBC transfusion on 90-day survival rates. The partial dependence plot revealed a nonlinear relationship between the 10 features included in the treatment effects of RBC transfusion (Figure 5). Patients with higher SAPS-II, WBC_min, BUN_max, Na_min, and RDW_min values generally experienced increased survival rates from RBC transfusion. Conversely, patients with higher HR_mean, PLT_min, Age, and HCO_3__min values were associated with decreased survival rates following RBC transfusion. RR_max and treatment effects exhibited an inverted U-shape relationship, where the survival rates initially increased and then decreased with an increase in RR_max. The ITE values for 90-day survival rates in the MIMIC-IV derivation cohort were: Quantile1: −1.1% (−2.4%, −0.3%), Quantile2: +1.0% (+0.1%, +1.4%), Quantile3: +2.6% (+2.1%, +3.3%), and Quantile4: +8.3% (+5.8%, +12.5%) (Supplementary Figure S6). In contrast, the ITE values on 90-day survival rates the MIMIC-III validation cohort were: Quantile1: −5.4% (−8.0%, −3.9%), Quantile2: −2.1% (−2.6%, −1.7%), Quantile3: −0.5% (−0.1%, +0.1%), and Quantile4: +3.6% (+2.0%, +6.6%) (Supplementary Figure S6). The Kaplan-Meier curves and log-rank test indicated that RBC transfusion decreased the patient survival rates in the Quantile1 subgroup (p < 0.01) but increased the survival rates in the Quantile4 subgroup (p < 0.01) in the MIMIC-IV derivation cohort (Supplementary Figure S7). RBC transfusion decreased the survival rates in Quantile1 (p < 0.001) and Quantile2 subgroups (p = 0.011) but increased the survival rates in the Quantile4 subgroup (p < 0.001) in the MIMIC-III validation cohort (Figure 6). In the MIMIC-IV database, the Quantile4 subgroup exhibited the highest values of SAPS II, WBC_min, BUN_max, PLT_min, RDW_min, age, and RR_max (all p < 0.05), as well as the lowest value of HCO_3__min (p < 0.05) (Supplementary Table S3). Similarly, in the MIMIC-III database, the Quantile4 subgroup demonstrated the highest SAPS II, WBC_min, BUN_max, PLT_min, RDW_min, age, and RR_max values (all p < 0.05), and the lowest HCO_3__min value (p < 0.05) (Supplementary Table S3).

## Discussion

Propensity score matching analysis revealed no significant differences in mortality risk between the RBC transfusion group and the non-RBC transfusion group of sepsis patients with Hb levels of 7–9 g/dL. Notably, the causal forest model suggested that RBC transfusion could reduce the mortality risk of patients with higher ITEs. The treatment efficacy was influenced by several parameters, including SAPS-II, WBC_min, BUN_max, Na_min, HR_mean, PLT_min, RDW_min, Age, RR_max, and HCO_3__min.

Microcirculatory dysfunction in sepsis patients results in inadequate tissue perfusion, leading to tissue and organ ischemia and hypoxia, subsequently impairing organ function. Anemia is a common complication in sepsis patients that decreases oxygen delivery, worsening organ failure (Jansma et al., 2015; Chan et al., 2017). The mortality risk in elderly sepsis patients progressively increases with a decrease in Hb levels (Yang et al., 2023). RBC transfusion enhances oxygen delivery, thereby improving tissue oxygenation in sepsis patients (Rivers et al., 2001). In contrast, Marik et al. demonstrated that increasing hemoglobin levels does not automatically translate into improved tissue oxygenation. Their study showed that even with sustained high hemoglobin concentrations (119 ± 9.0 g/L), patients did not experience a meaningful enhancement in oxygen supply to tissues. Noteworthy, there was a decrease in gastric intramucosal pH (Marik and Sibbald, 1993). This report was similar to that of Fernandes et al., who postulated that RBC transfusion did not significantly increase global or regional oxygen utilization (Fernandes et al., 2001). These reports collectively suggest that administering RBC blood products to patients with high Hb concentrations confers no significant therapeutic advantage or improvement in clinical outcomes.

The 2016 and 2021 Surviving Sepsis Campaign guidelines based on the TRISS and TRICC studies recommend a restrictive transfusion strategy for managing RBC transfusions in sepsis patients (Hébert et al., 1999; Holst et al., 2014; Rhodes et al., 2017). In both studies, patients transfused at a threshold of 7 g/dL and 9 g/dL had insignificant differences in mortality. However, there was potentially significant heterogeneity in response across different patient subpopulations (Hébert et al., 1999; Holst et al., 2014; Rhodes et al., 2017). A retrospective study by Nilsson et al. reported that low-dose transfusion in sepsis patients potentially increases the mortality risk (Nilsson et al., 2020). Nonetheless, the patients in Nilsson’s study had a high Hb level of 9.5 g/dL, and the study did not determine whether blood transfusion benefited sepsis patients with Hb levels between 7–9 g/dL (Nilsson et al., 2020). A transfusion threshold of 9 g/dL was associated with reduced mortality risk in a survey of liberal versus restrictive transfusion strategies in critically ill oncologic patients (Bergamin et al., 2017). Chen et al. reported that blood transfusion potentially reduces the mortality risk in sepsis patients with CKD (Chen et al., 2024). These reports suggest that sepsis patients with Hb levels of 7–9 g/dL may benefit from RBC transfusion depending on the severity of their condition and underlying diseases.

Causal forest is a tree-based ensemble method designed to estimate heterogeneous treatment effects. In contrast to T-learner and S-learner methods, which require fitting separate or combined models for potential outcomes, causal forests are designed to directly estimate individual treatment effects. They inherently accommodate complex nonlinear interactions and high-order feature dependencies without the need for strong parametric assumptions. This makes causal forests particularly suitable for settings with complex and unknown effect heterogeneity (Athey and Imbens, 2016; Tibshirani et al., 2024; Xie et al., 2024).

The causal forest model uncovered high heterogeneity in the ITEs of RBC transfusion among sepsis patients with Hb levels of 7–9 g/dL. The treatment effect of transfusion on 90-day survival was affected by factors such as SAPS-II, WBC, BUN, Na, HR, PLT, RDW, age, RR, and HCO_3_. In the Quantile4 subgroup, which benefited from transfusion, the patients showed higher SAPS II, WBC_min, BUN_max, PLT_min, RDW_min, age, and RR_max values, and the lowest HCO_3__min. The partial dependence plot indicated that patients with high SAPS-II scores and elevated WBC, BUN, Na, and RDW levels benefited from RBC transfusions and exhibited increased survival rates. These patients represented a subgroup likely to benefit from transfusion, as they generally presented with more severe clinical conditions and a higher risk of mortality. Elevated SAPS II scores, along with increased levels of WBC, BUN, sodium, and RDW, are indicative of inadequate tissue perfusion and severe ischemia-hypoxia. Red blood cell transfusion may enhance oxygen delivery, thereby alleviating tissue ischemia and hypoxia and potentially improving clinical outcomes (Poncet et al., 2017; Cavalcante Dos Santos et al., 2020).

Although the Quantile4 subgroup had higher PLT, analysis of the partial dependence plot showed that excessively elevated platelet levels correlated with increased risk of mortality. This discrepancy suggests that, although univariate analysis demonstrated higher PLT in the Quantile4 subgroup, the partial dependence plot confirmed the independent association between PLT and mortality after adjusting for other covariates. Therefore, the partial dependence plot provides a more nuanced assessment of the relationship between PLT and mortality risk by adjusting for potential confounders. On the other hand, patients with higher PLT counts have a higher risk of thrombosis. Of note, RBC transfusion can exacerbate PLT activation, worsening microcirculatory thrombosis (Czubak-Prowizor et al., 2020). In septic patients, increased tissue ischemia and hypoxia can lead to the accumulation of acidic metabolites, resulting in decreased HCO_3_^−^ levels. Since red blood cell transfusion enhances oxygen-carrying capacity, its benefits are more likely to be observed in patients with impaired tissue oxygenation. In contrast, patients with normal HCO_3_^−^ levels—who are less likely to experience significant tissue ischemia or hypoxia—may derive limited benefit from transfusion. In such cases, the potential risks of transfusion, including fluid overload, transfusion-related inflammation, acute respiratory distress syndrome (ARDS), and acute kidney injury (AKI), may outweigh any marginal benefits, potentially leading to net harm. RR and transfusion benefits have an inverted U-shape relationship where the survival impact of transfusion initially increases and then decreases with an increase in RR. Patients with excessively high RR often experience complications, such as volume overload and severe ARDS. Of note, RBC transfusions might exacerbate these conditions, increasing their mortality risk. TOC curves and AUTOC values observed in this study suggest meaningful individual variability in the benefit of low-dose RBC transfusions among septic patients with hemoglobin levels between 7 and 9 g/dL. Patients with higher predicted benefit scores experienced a greater reduction in mortality risk following transfusion. Though the causal forest model may effectively guide personalized transfusion strategies, further randomized controlled trials are needed to confirm these findings.

### Limitations

This study was limited by several factors. 1) The study used retrospective data, and the findings should thus be validated using prospective randomized controlled trials. 2) The model used did not include CRP, PCT, and D-dimer markers because of the high number of missing values. The absence of these inflammatory and thrombosis-related markers could have potentially influenced the effects of transfusion.

## Conclusion

RBC transfusions among sepsis patients with Hb levels of 7–9 g/dL exhibit heterogeneity of treatment effects, potentially reducing the mortality of patients with high ITE. Moreover, the treatment effect of RBC transfusion on 90-day survival is influenced by multiple factors, such as SAPS-II, WBC, BUN, Na, HR, PLT, RDW, age, RR, and HCO_3_. Although the causal forest model can guide personalized transfusion, randomized controlled trials are advocated to further validate the present results.

## Data Availability

The raw data supporting the conclusions of this article will be made available by the authors, without undue reservation.

## Abbreviations

RBC, Red Blood Cell; ITE, Individual Treatment Effect; ATE, Average Treatment Effect; Hb, Hemoglobin; MIMIC, Medical Information Mart for Intensive Care; SMD, Standardized Mean Difference; HR, Heart Rate; MBP, Mean Blood Pressure; RR, Respiratory Rate; T, Temperature; SPO2, Oxygen Saturation; G, Glucose; WBC, White Blood Cell; BUN, Blood Urea Nitrogen; Na, Sodium; K, Potassium; PT, Prothrombin Time; APTT, Activated Partial Thromboplastin Time; VDI_24hmax, Maximum Norepinephrine Equivalent dose within 24 h in ICU; Lac, Lactate; PO2, Arterial Oxygen Partial Pressure; Fluid_6h_sum, Volume of intravenous infusion within six h of ICU admission; Fluid_24h_sum, Volume of intravenous infusion within 24h of ICU admission; SAPS-II, Simplified Acute Physiology Score II; SAPS-III, Simplified Acute Physiology Score III; SIRS, Systemic Inflammatory Response Syndrome; Charlson, Charlson comorbidity index; SOFA, Sequential Organ Failure Assessment; CRD, Chronic Renal Disease. Venti, Mechanical Ventilation. TOC, Targeting Operator Characteristic; AUTOC, the Area Under the Targeting Operator Characteristic.
