# Supplementary material for: Investigating the feasibility of machine learning to guide personalized red blood cell (RBC) transfusion: analyzing the heterogeneity of RBC transfusion in septic patients with hemoglobin levels of 7–9 g/dL based on the causal forest model
Source: Front Pharmacol. 2025 Aug 28;16:1615618. doi: 10.3389/fphar.2025.1615618 (PMC12423935; doi:10.3389/fphar.2025.1615618)
Supplement: Supplementary file 1 [file Supplementaryfile1.docx]

**Supplemental Material: Investigating the Feasibility of Machine Learning to Guide Personalized Red Blood Cell (RBC) Transfusion: Analyzing the Heterogeneity of RBC Transfusion in Septic Patients with Hemoglobin Levels of 7-9 g/dL Based on the Causal Forest Model**

**1.Methods1**

Appendix A. Definition of Sepsis1

Appendix B. The screening process of patients with trauma, acute myocardial infarction, and gastrointestinal bleeding.1

Appendix C. Patient data extracted for the study 4

Appendix D. Imputation methods for missing data.9

Appendix E. R code for statistical analysis.28

**2.Results11**

Table S1. Baseline characteristics of RBC transfusion and non-transfusion groups in the MIMIC-IV and MIMIC-III database 11

Appendix E. Variable selection using Lasso regression and Boruta algorithm 17

Figure S1. Kaplan-Meier curves for the RBC transfusion and non-transfusion groups in MIMIC-IV and MIMIC-III after propensity score matching 20

Table S2. Hemoglobin levels in the RBC transfusion and non-transfusion groups in the MIMIC-IV and MIMIC-III database after propensity score matching.21

Figure S2. Ridgeline plot of hemoglobin levels across different time frames 22

Figure S3. Hemoglobin trajectory classification plot 23

Figure S4. Individual hemoglobin trajectories in the MIMIC-IV and MIMIC-III database after propensity score matching 23

Figure S5 Targeting Operator Characteristic (TOC) curve for evaluating the MIMIC−III validation cohort23

Figure S6 Individual treatment effects (ITEs) on 90-day survival rates for patient subgroups across various quantiles 24

Figure S7. The Kaplan-Meier curves for patients in Quantiles 1 to 4 subgroups of the MIMIC-IV derivation cohort 25

TableS3. Differences in baseline characteristics among the different quantile subgroups. 26

**1. Methods**

**Appendix A .** **Definition of Sepsis.**

According to the Surviving Sepsis Campaign 2021 guidelines, the diagnosis of sepsis is based on the Sepsis-3 criteria, which require the presence of an infection and a SOFA score ≥ 2 (Evans et al., 2021). Clinical Suspicion of Infection in the MIMIC Database: This is determined based on the timing of antibiotic administration and cultures performed within a specific timeframe. If antibiotics are administered first, cultures must be performed within 24 hours. If cultures are performed first, antibiotics must be administered within 72 hours. Organ Dysfunction: Identified by a decrease in the SOFA score by two points. Onset Time of Sepsis: The onset time of sepsis is the earlier of $t_{\mathrm{suspicion}}$ and $t_{\mathrm{SOFA}}$ as long as $t_{\mathrm{SOFA}}$ occurs no more than 48 hours before or 24 hours after $t_{\mathrm{suspicion}}$; otherwise, the patient is not marked as a sepsis patient. Specifically, if $t_{\mathrm{suspicion}}$ -48 ≤ $t_{\mathrm{SOFA}}$ ≤ $t_{\mathrm{suspicion}}$ +24, then $t_{\mathrm{sepsis}}$ = min($t_{\mathrm{suspicion}}$, $t_{\mathrm{SOFA}}$) (Hu et al., 2023). Sepsis Extraction is Similar to Previous Studies (Seymour et al., 2016;Reyna et al., 2020;Yang et al., 2020;Hu et al., 2023).

**Appendix B. The screening process of patients with trauma, acute myocardial infarction, and gastrointestinal bleeding.**

Due to the significant differences in pathophysiological mechanisms and treatment approaches between patients with combined trauma, acute myocardial infarction, gastrointestinal bleeding, and those with uncomplicated sepsis, this study excluded these patients based on ICD-9 and ICD-10 codes.

**ICD-9 Codes for Trauma:**

| ICD-9 Codes for Trauma | Category | ICD-9 codes |
| --- | --- | --- |
|  | Skull Fractures | 800-804 |
|  | Fractures of the Neck, Spine, and Trunk | 805-809 |
|  | Fractures of the Upper Limb | 810-819 |
|  | Fractures of the Lower Limb | 820-829 |
|  | Dislocations | 830-839 |
|  | Sprains and Strains of Joints and Adjacent Muscles | 840-848 |
|  | Intracranial Injuries, Excluding Those with Skull Fracture | 850-854 |
|  | Internal Injuries of Chest, Abdomen, and Pelvis | 860-869 |
|  | Open Wounds of Head, Neck, and Trunk | 870-879 |
|  | Open Wounds of the Upper Limb | 880-887 |
|  | Open Wounds of Lower Limb | 890-897 |
|  | Blood Vessel Injuries | 900-904 |
|  | Crushing Injuries | 925-929 |
|  | Burns | 940-949 |
|  | Injuries to Nerves and Spinal Cord | 950-957 |

**ICD-10 Codes for Trauma:**

| ICD-10 Codes for Trauma | Category | ICD-10 codes |
| --- | --- | --- |
|  | Head Injuries | S00-S09 |
|  | Neck Injuries | S10-S19 |
|  | Thoracic Injuries | S20-S29 |
|  | Injuries to Abdomen, Lower Back, Lumbar Spine, Pelvis, and External Genitals | S30-S39 |
|  | Shoulder and Upper Arm Injuries | S40-S49 |
|  | Elbow and Forearm Injuries | S50-S59 |
|  | Wrist, Hand, and Finger Injuries | S60-S69 |
|  | Hip and Thigh Injuries | S70-S79 |
|  | Knee and Lower Leg Injuries | S80-S89 |
|  | Ankle and Foot Injuries | S90-S99 |
|  | Unspecified Multiple Injuries | T07 |
|  | Injuries of Unspecified Body Region | T14 |
|  | Burns and Corrosions of External Body Surface, Specified by Site | T20-T25 |
|  | Burns and Corrosions Confined to the Eye and Internal Organs | T26-T28 |
|  | Burns and Corrosions of Multiple and Unspecified Body Regions | T30-T32 |

**ICD Codes for Gastrointestinal Bleeding:**

| ICD Codes for Gastrointestinal Bleeding | ICD-9 codes | ICD-10 codes |
| --- | --- | --- |
|  | 53021, 5307, 53082, 53100, 53101, 53120, 53121, 53200, 53201, 53221, 53300, 53321, 53301, 53400, 53401, 53420, 53501, 53521, 53531, 53541, 53561, 53571, 53511, 53551, 5780, 5781, 5789 | K2211, K250, K260, K262, K272, K280, K282, K2901, K2991, K2981, K2971, K2961, K2951, K2941, K2931, K2921, K625, K661 |

**ICD-10 Codes for Acute Myocardial Infarction:**

| ICD-10 Codes for Acute Myocardial Infarction | ICD-9 codes | ICD-10 codes |
| --- | --- | --- |
|  | 41000, 41001, 41002, 41010, 41011, 41012, 41020, 41021, 41022, 41030, 41031, 41032, 41040, 41041, 41042, 41050, 41051, 41052, 41060, 41061, 41062, 41070, 41071, 41072, 41080, 41081, 41082, 41090, 41091, 41092 | I22, I220, I221, I222, I228, I229, I21, I210, I2101, I2102, I2109, I211, I2111, I2119, I212, I2121, I2129, I213, I214, I219, I21A, I21A1, I21A9, I23, I230, I231, I232, I233, I234, I235, I236, I237, I238 |

**Appendix C. Patient data extracted for the study.**

This study extracted characteristics, including demographic information, vital signs, blood gas analysis, liver and kidney function, coagulation function, comorbidities, disease severity scores, and the use of vasoactive drugs.

| **Indicator** | **Description** | **Unit** |
| --- | --- | --- |
| Gender | Male | n(%) |
| Age | Age at admission | Year |
| Weight | Weight at admission | Kg |
| Height | Height at admission | m |
| HR_max | Maximum heart rate within 24 hours in ICU | bpm |
| HR_mean | Mean heart rate within 24 hours in ICU | bpm |
| MBP_min | Minimum mean blood pressure within 24 hours in ICU | mmHg |
| MBP_mean | Mean blood pressure within 24 hours in ICU | mmHg |
| RR_max | Maximum respiratory rate within 24 hours in ICU | bpm |
| RR_mean | Mean respiratory rate within 24 hours in ICU | bpm |
| T_min | Minimum temperature within 24 hours in ICU | °C |
| T_max | Maximum temperature within 24 hours in ICU | °C |
| T_mean | The mean temperature within 24 hours in ICU | °C |
| SPO_2__min | Minimum oxygen saturation within 24 hours in ICU | % |
| SPO_2__mean | Mean oxygen saturation within 24 hours in ICU | % |
| G_min | Minimum glucose within 24 hours in ICU | mg/dL |
| G_max | Maximum glucose within 24 hours in ICU | mg/dL |
| G_mean | Mean glucose within 24 hours in ICU | mg/dL |
| ad_Hb | Hemoglobin at admission ICU | g/dL |
| Hb_min | Minimum hemoglobin levels during the ICU stay. | g/dL |
| Hb_max | Maximum hemoglobin levels during the ICU stay. | g/dL |
| Hb_1d_min | Minimum hemoglobin level on the first day of ICU admission | g/dL |
| Hb_1d_max | Maximum hemoglobin level on the first day of ICU admission | g/dL |
| Hb_2d_min | Minimum hemoglobin level on the secondary day of ICU admission | g/dL |
| Hb_2d_max | Maximum hemoglobin level on the secondary day of ICU admission | g/dL |
| Hb_3d_min | Minimum hemoglobin level on the third day of ICU admission | g/dL |
| Hb_3d_max | Maximum hemoglobin level on the third day of ICU admission | g/dL |
| Hb_4d_min | Minimum hemoglobin level on the fourth day of ICU admission | g/dL |
| Hb_4d_max | Maximum hemoglobin level on the fourth day of ICU admission | g/dL |
| Hb_5d_min | Minimum hemoglobin level on the fifth day of ICU admission | g/dL |
| Hb_5d_max | Maximum hemoglobin level on the fifth day of ICU admission | g/dL |
| PLT_min | Minimum platelet count within 24 hours in ICU | 10^9^/L |
| WBC_min | Minimum white blood cell count within 24 hours in ICU | 10^9^/L |
| WBC_max | Maximum white blood cell count within 24 hours in ICU | 10^9^/L |
| Albumin_min | Minimum albumin within 24 hours in ICU | g/L |
| HCO3_min | Minimum bicarbonate within 24 hours in ICU | mmol/L |
| BUN_max | Maximum blood urea nitrogen within 24 hours in ICU | mmol/L |
| Ca_min | Minimum calcium within 24 hours in ICU | mmol/L |
| Cr_max | Maximum creatinine within 24 hours in ICU | mg/dL |
| Na_min | Minimum sodium within 24 hours in ICU | mmol/L |
| Na_max | Maximum sodium within 24 hours in ICU | mmol/L |
| K_max | Maximum potassium within 24 hours in ICU | mmol/L |
| LYM_min | Minimum lymphocyte count within 24 hours in ICU | 10^9^/L |
| D_Dimer_max | Maximum D-Dimer within 24 hours in ICU | mg/L |
| Fib_min | Minimum fibrinogen within 24 hours in ICU | g/L |
| Fib_max | Maximum fibrinogen within 24 hours in ICU | g/L |
| INR_max | Maximum international normalized ratio within 24 hours in ICU | - |
| PT_max | Maximum prothrombin time within 24 hours in ICU | s |
| APTT_max | Maximum activated partial thromboplastin time within 24 hours in ICU | s |
| ALT_max | Maximum alanine aminotransferase within 24 hours in ICU | U/L |
| AST_max | Maximum aspartate aminotransferase within 24 hours in ICU | U/L |
| TBIL_max | Maximum total bilirubin within 24 hours in ICU | mg/dL |
| Venti | Use of mechanical ventilation | n(%) |
| AKI | Occurrence of acute kidney injury | n(%) |
| VDI_24hmax | Maximum Norepinephrine Equivalent dose within 24 hours in ICU | μg/kg/min |
| Lac_max | Maximum lactate within 24 hours in ICU | mmol/L |
| PH_min | Minimum blood pH within 24 hours in ICU | - |
| PO_2__min | Minimum arterial oxygen partial pressure within 24 hours in ICU | mmHg |
| PCO_2__max | Maximum arterial carbon dioxide partial pressure within 24 hours in ICU | mmHg |
| P/F_min | Minimum Arterial Oxygen Partial Pressure / Fractional Inspired Oxygen within 24 hours in ICU | mmHg |
| CHF | Congestive heart failure | n(%) |
| CVD | Cerebrovascular disease | n(%) |
| CPD | Chronic pulmonary disease | n(%) |
| RD | Rheumatic disease | n(%) |
| CRD | Chronic renal disease | n(%) |
| Cancer | Cancer | n(%) |
| Charlson | Charlson comorbidity index | n(%) |
| SAPS_II | Simplified Acute Physiology Score II | n(%) |
| SAPS_III | Simplified Acute Physiology Score III | n(%) |
| SIRS | Systemic Inflammatory Response Syndrome | n(%) |
| SOFA | Sequential Organ Failure Assessment | n(%) |
| RBC_trans | Red blood cell transfusion | n(%) |
| Fluid_3h_sum | Volume of intravenous infusion within three hours of ICU admission | ml |
| Fluid_6h_sum | Volume of intravenous infusion within six hours of ICU admission | ml |
| Fluid_24h_sum | Volume of intravenous infusion within 24h of ICU admission | ml |
| RDW_min | Minimum Red Cell Distribution Width (RDW) within the first 24 hours of ICU admission | % |
| RDW_max | Maximum Red Cell Distribution Width (RDW) within the first 24 hours of ICU admission | % |
| Type of ICU | MICU、M-SICU、SICU、CICU | n(%) |
| Anemia | Chronic anemia | n(%) |
| Survival_90d | 90-day survival rate | n(%) |

**Appendix D. Imputation methods for missing data.**

There is no consensus on the standard percentage of missing values for excluding variables in the analysis. Hu et al. (Hu et al., 2023) retained features with less than 60% missing values in their study, while Zhang et al. (Zhang et al., 2019)retained variables with less than 70% missing values. In our study, we used a threshold of 40% for missing values. Variables with more than 40% missing values were excluded. We assessed whether the missing values were Missing at Random (MAR). We used the multiple imputation method with the “mice”(v4.4.3) package in R to imputation the missing variables. The figure below shows the top 20 missing data rankings for patient characteristics in the MIMIC-IV and MIMIC-III datasets.

**
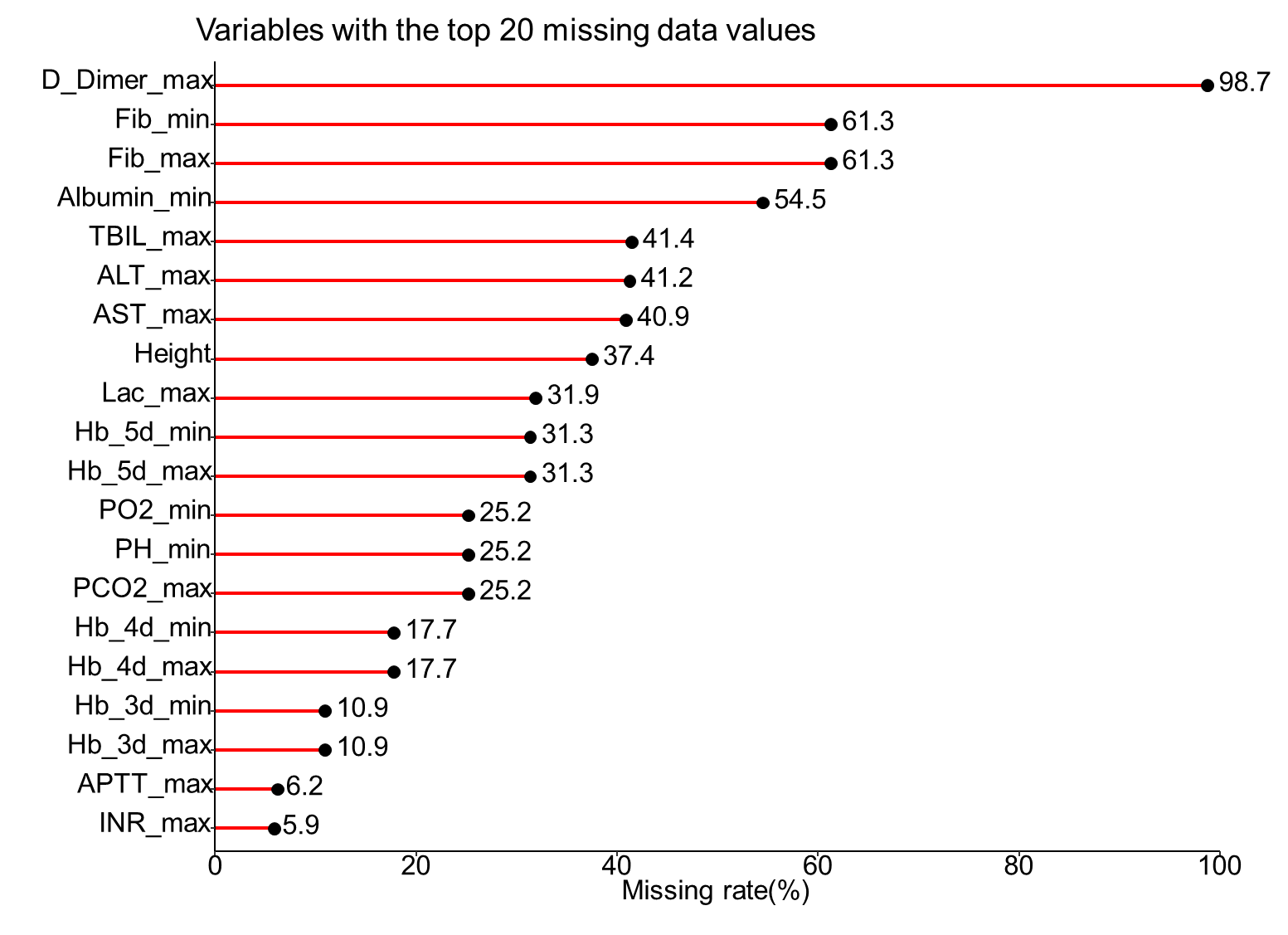
**

**Top 20 Missing Data Rankings for Patient Characteristics in the MIMIC-IV Dataset.** Fib: Fibrinogen; TBIL: Total Bilirubin; ALT: Alanine Aminotransferase; AST: Aspartate Aminotransferase; Lac: Lactate; Hb: hemoglobin; PO_2_: Arterial Oxygen Partial Pressure; PCO_2_: Arterial Carbon Dioxide Partial Pressure; APTT: Activated Partial Thromboplastin Time; INR: International Normalized Ratio.


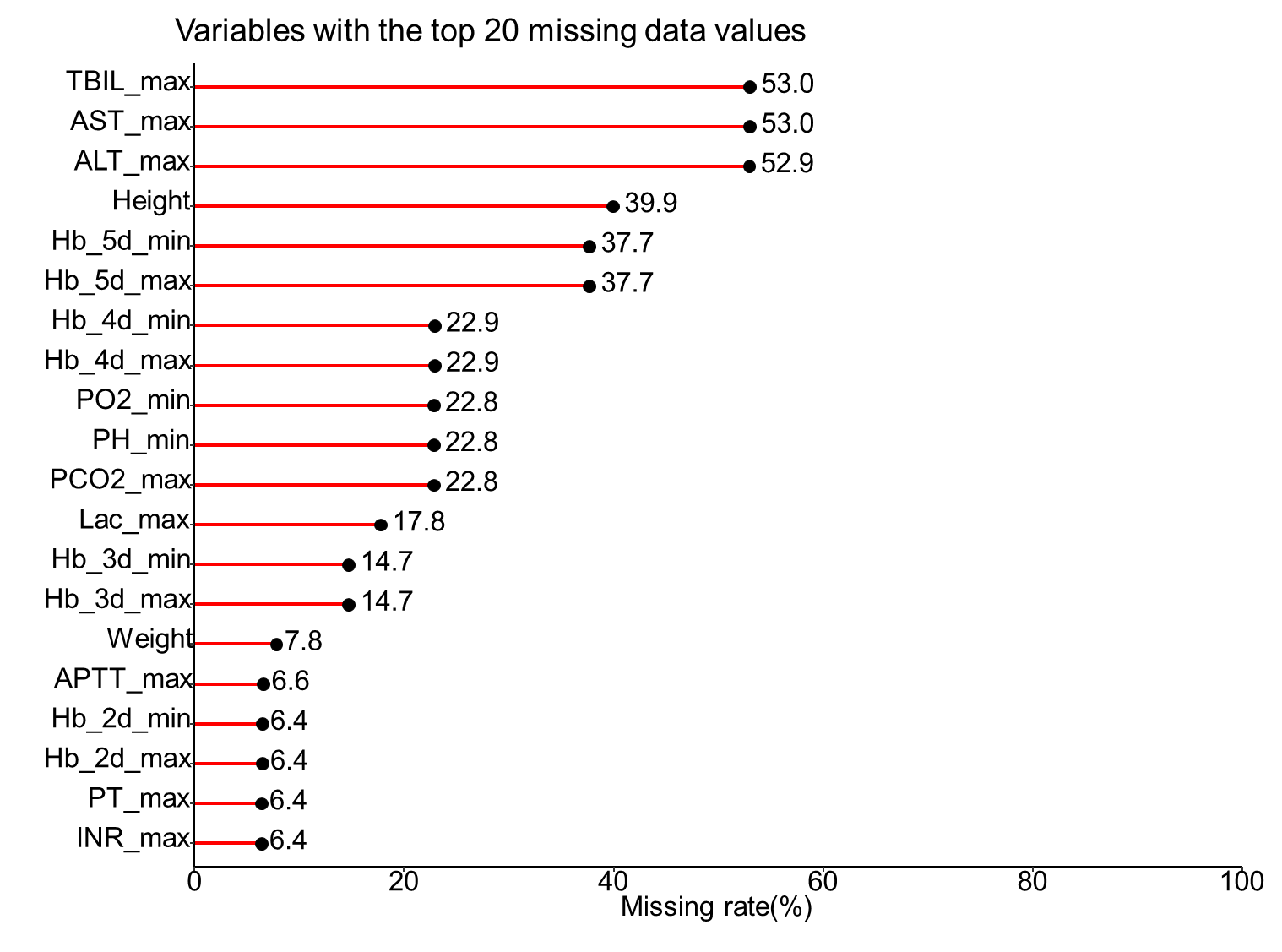


**Top 20 Missing Data Rankings for Patient Characteristics in the MIMIC-III Dataset.** TBIL: Total Bilirubin; AST: Aspartate Aminotransferase; ALT: Alanine Aminotransferase; Hb: hemoglobin; PO_2_: Arterial Oxygen Partial Pressure; PCO_2_: Arterial Carbon Dioxide Partial Pressure; Lac: Lactate; APTT: Activated Partial Thromboplastin Time; PT: Prothrombin Time; INR: International Normalized Ratio.

**2. Results**

**Table S1** Baseline characteristics of RBC transfusion and non-transfusion groups in the MIMIC-IV and MIMIC-III database.

| **Variable** | MIMIC-IV | | | MIMIC-III | | |
| --- | --- | --- | --- | --- | --- | --- |
|  | Non-transfusion  *N=5325* | RBC transfusion  *N=857* | p | Non-transfusion  *N=1882* | RBC transfusion  *N=458* | p |
| Male(n%) | 2610(49.01%) | 390(45.51%) | 0.062 | 929(49.36%) | 204(44.54%) | 0.072 |
| Age(years) | 68.70[58.48;78.85] | 69.65[59.65;79.78] | 0.061 | 67.66[57.13;77.00] | 69.56[57.55;79.47] | 0.015 |
| Weight(Kg) | 78.30[65.70;93.00] | 76.50[65.00;90.75] | 0.073 | 78.00[66.00;92.00] | 75.00[63.53;87.50] | 0.003 |
| HR_max(bpm) | 103.00[90.00;117.00] | 101.00[89.00;115.00] | 0.023 | 103.00[91.00;117.00] | 103.50[91.00;118.00] | 0.600 |
| HR_mean(bpm) | 85.09[75.79;96.66] | 83.57[75.97;94.97] | 0.247 | 86.32[77.85;95.96] | 87.04[79.23;97.21] | 0.051 |
| MBP_min(mmHg) | 57.00[51.00;63.00] | 56.00[49.00;61.00] | <0.001 | 56.00[50.00;62.00] | 55.00[49.00;60.00] | 0.026 |
| MBP_mean(mmHg) | 74.26[69.32;80.40] | 72.64[68.92;77.64] | <0.001 | 74.02[68.84;79.80] | 72.57[67.51;78.43] | 0.004 |
| RR_max | 28.00[24.00;32.00] | 26.00[23.00;30.00] | <0.001 | 27.00[24.00;31.00] | 26.00[23.00;30.00] | 0.052 |
| RR_mim | 18.96[16.70;21.90] | 17.88[16.00;20.29] | <0.001 | 18.46[16.27;21.27] | 18.03[15.69;20.82] | 0.024 |
| T_min(℃) | 36.44[36.00;36.67] | 36.33[35.67;36.56] | <0.001 | 36.10[35.61;36.50] | 35.83[35.46;36.43] | <0.001 |
| T_max(℃) | 37.28[37.00;37.83] | 37.33[37.00;37.83] | 0.064 | 37.50[37.00;38.10] | 37.56[37.00;38.06] | 1.00 |
| T_mean(℃) | 36.83[36.58;37.14] | 36.79[36.54;37.07] | 0.010 | 36.81[36.45;37.21] | 36.78[36.40;37.16] | 0.127 |
| SPO_2__min(%) | 93.00[90.00;95.00] | 93.00[91.00;95.00] | 0.004 | 93.00[90.00;95.00] | 93.00[90.00;95.00] | 0.802 |
| SPO_2__mean(%) | 97.24[95.85;98.46] | 97.77[96.55;98.81] | <0.001 | 97.52[96.21;98.57] | 97.72[96.51;98.73] | 0.031 |
| G_min(mg/dL) | 99.00[84.00;122.00] | 96.00[82.00;117.00] | 0.001 | 96.00[82.00;116.00] | 90.00[79.00;110.00] | <0.001 |
| G_max(mg/dL) | 166.00[133.00;209.00] | 170.00[145.00;208.00] | 0.009 | 168.00[139.00;203.00] | 169.00[140.00;202.75] | 0.797 |
| G_mean(mg/dL) | 129.67[113.00;154.28] | 128.75[116.68;149.43] | 0.956 | 127.72[113.93;149.08] | 124.69[113.35;139.14] | 0.040 |
| PLT_min(10^9^/L) | 162.00[112.00;239.00] | 131.00[96.00;189.00] | <0.001 | 167.00[116.00;251.00] | 135.50[98.50;214.00] | <0.001 |
| PLT_max(10^9^/L) | 201.00[143.00;285.00] | 176.00[130.00;247.00] | <0.001 | 206.00[148.25;301.00] | 179.00[131.00;256.75] | <0.001 |
| WBC_min(10^9^/L) | 9.80[6.80;13.60] | 9.10[6.40;12.30] | <0.001 | 9.80[6.60;13.30] | 8.80[6.40;12.50] | 0.007 |
| WBC_max(10^9^/L) | 13.80[9.70;18.70] | 13.70[10.20;18.20] | 0.846 | 13.10[9.50;18.00] | 12.900[9.425;18.00] | 0.605 |
| HCT_min | 27.30[25.20;29.60] | 24.20[22.90;26.0] | <0.001 | 26.30[24.00;28.50] | 23.50[22.00;25.575] | <0.001 |
| HCT_max | 31.50[28.90;34.60] | 30.30[28.00;32.90] | <0.001 | 32.60[29.30;36.40] | 32.00[28.43;35.00] | 0.004 |
| HCO_3__min | 21.00[18.00;24.00] | 22.00[19.00;24.00] | 0.731 | 22.00[20.00;25.00] | 22.00[20.00;24.00] | 0.076 |
| BUN_min(mg/dL) | 19.00[13.00;33.00] | 18.00[12.00;28.00] | 0.009 | 18.00[12.00;31.00] | 18.00[12.00;29.00] | 0.770 |
| BUN_max(mg/dL) | 23.00[15.00;40.00] | 21.00[14.00;35.00] | 0.001 | 21.00[15.00;37.00] | 21.00[15.00;34.750] | 0.291 |
| Cr_max(mg/dL) | 1.10[0.80;1.90] | 1.10[0.80;1.60] | <0.001 | 1.10[0.80;1.80] | 1.00[0.80;1.60] | 0.101 |
| Cr_min(mg/dL) | 1.00[0.70;1.50] | 0.90[0.60;1.30] | <0.001 | 0.90[0.70;1.50] | 0.90[0.60;1.30] | 0.042 |
| Na_min(mmol/L) | 137.00[134.00;139.00] | 137.00[134.00;139.00] | 0.726 | 136.00[134.00;138.00] | 135.00[133.00;137.00] | <0.001 |
| Na_max(mmol/L) | 139.00[137.00;142.00] | 140.00[137.00;142.00] | 0.693 | 140.00[137.00;142.00] | 140.00[138.00;142.00] | 0.782 |
| K_max(mmol/L) | 4.50[4.10;4.90] | 4.50[4.10;4.90] | 0.686 | 4.70[4.20;5.30] | 4.80[4.20;5.48] | 0.093 |
| INR_max | 1.40[1.20;1.60] | 1.40[1.30;1.70] | <0.001 | 1.40[1.20;1.60] | 1.40[1.30;1.70] | <0.001 |
| PT_max(s) | 15.20[13.40;17.90] | 15.60[14.10;18.40] | <0.001 | 15.00[13.80;17.10] | 15.70[14.50;17.68] | <0.001 |
| APTT_max(s) | 32.70[28.60;41.10] | 34.70[30.00;44.50] | <0.001 | 34.10[29.00;41.90] | 37.40[31.23;47.08] | <0.001 |
| Lac_max(s) | 2.00[1.40;3.10] | 2.40[1.60;3.60] | <0.001 | 2.20[1.50;3.10] | 2.40[1.60;3.58] | 0.001 |
| PH_min | 7.34[7.27;7.39] | 7.33[7.28;7.37] | 0.018 | 7.34[7.28;7.39] | 7.33[7.28;7.38] | 0.198 |
| PO2_min(mmHg) | 85.00[64.00;116.00] | 90.00[72.00;121.00] | <0.001 | 89.00[71.00;118.00] | 88.00[72.00;114.00] | 0.778 |
| PCO_2__max(mmHg) | 45.00[38.00;52.00] | 45.00[40.00;50.00] | 0.809 | 45.50[39.00;52.00] | 46.00[40.00;51.00] | 0.715 |
| Charlson | 6.00[4.00;8.00] | 6.00[4.00;7.00] | 0.004 | 5.00[3.00;7.00] | 5.00[3.00;6.00] | 0.681 |
| SOFA | 3.00[2.00;4.00] | 3.00[2.00;5.00] | 0.012 | 5.00[3.00;7.00] | 6.00[4.00;8.00] | <0.001 |
| SAPS_II | 38.00[31.00;48.00] | 39.00[31.00;49.00] | 0.229 | 37.00[30.00;47.00] | 38.00[31.00;47.00] | 0.095 |
| SAPS_III | 49.00[36.00;67.00] | 46.00[33.00;65.00] | <0.001 | 45.00[34.00;61.00] | 45.00[33.00;61.00] | 0.875 |
| SIRS | 3.00[2.00;3.00] | 3.00[2.00;3.00] | 0.111 | 3.00[2.00;4.00] | 3.00[2.00;4.00] | 0.564 |
| RDW_min(%) | 14.70[13.60;16.50] | 14.70[13.60;16.30] | 0.467 | 14.80[13.70;16.38] | 14.85[13.80;16.60] | 0.211 |
| RDW_max(%) | 15.10[13.90;16.80] | 15.20[14.20;17.00] | 0.003 | 15.20[14.00;16.80] | 15.40[14.325;17.20] | <0.001 |
| Hb_min(g/dL) | 8.20[7.70;8.60] | 7.70[7.30;8.20] | <0.001 | 8.40[7.90;8.70] | 8.00[7.50;8.40] | <0.001 |
| Hb_max(g/dL) | 10.30[9.50;11.30] | 10.30[9.60;11.10] | 0.755 | 10.40[9.70;11.20] | 10.50[9.70;11.20] | 0.930 |
| VDI_24h_max  (µg/min) | 0.00[0.00;0.10] | 0.06[0.00;0.15] | <0.001 | 0.00[0.00;0.08] | 0.05[0.00;0.15] | <0.001 |
| Fluid_3h_sum(ml) | 45.37[0;412] | 173[0;940] | <0.001 | 58[0;971] | 164[0;1005] | 0.009 |
| Fluid_6h_sum(ml) | 524 [55;1316] | 1082 [402;2235] | <0.001 | 860.50[104;1794] | 1065 [241;2481] | <0.001 |
| Fluid_24h_sum(ml) | 2242 [700;3696] | 3607 [2195;5138] | <0.001 | 2205 [784;3760.] | 3120 [1020;4493] | <0.001 |
| Shock(%) | 2484(46.65%) | 574(66.98%) | <0.001 | 852(45.27%) | 294(64.192%) | <0.001 |
| Anemia(%) | 2026(38.05%) | 297(34.66%) | 0.062 | 531(28.23%) | 133(29.04%) | 0.769 |
| CHF(%) | 1464(27.49%) | 195(22.75%) | 0.004 | 506(26.89%) | 135(29.48%) | 0.291 |
| CVD(%) | 658(12.36%) | 79(9.22%) | 0.010 | 141(7.49%) | 30(6.55%) | 0.552 |
| CPD(%) | 1408(26.44%) | 235(27.42%) | 0.575 | 475(25.24%) | 109(23.80%) | 0.563 |
| RD(%) | 247(4.64%) | 38(4.43%) | 0.859 | 87(4.62%) | 13(2.84%) | 0.118 |
| CRD(%) | 1381(25.93%) | 167(19.49%) | <0.001 | 426(22.64%) | 83(18.12%) | 0.042 |
| Cancer(%) | 976(18.33%) | 115(13.42%) | 0.001 | 299(15.89%) | 77(16.81%) | 0.680 |
| Type of ICU(%) | | | | | | |
| MICU | 1302(24.45%) | 138(16.103%) | <0.001 | 827(43.94%) | 165(36.03%) | <0.001 |
| M_SICU | 1308(24.56%) | 150(17.50%) |  | - | - |  |
| SICU | 1073(20.15%) | 171(19.95%) |  | 358(19.02%) | 76(16.59%) |  |
| CICU | 1642(30.84%) | 398(46.44%) |  | 697(37.04%) | 217(47.38%) |  |
| Source of infection(%) | | | | | | |
| Lung | 670(12.58%) | 65(7.59%) | <0.001 | 208(11.05%) | 47(10.26%) | 0.171 |
| Abdomen | 208(3.91%) | 29(3.38%) |  | 101(5.37%) | 17(3.71%) |  |
| Urinary | 430(8.08%) | 72(8.40%) |  | 219(11.64%) | 42(9.17%) |  |
| Blood | 1240(23.29%) | 142(16.57%) |  | 254(13.50%) | 74(16.16%) |  |
| Other | 2777(52.15%) | 549(64.06%) |  | 1100(58.45%) | 278(60.70%) |  |
| MV(%) | 2465(46.29%) | 503(58.69%) | <0.001 | 1130(60.04%) | 314(68.56%) | 0.001 |
| Death_90d(%) | 1351(25.37%) | 154(17.97%) | <0.001 | 360(19.13%) | 101(22.05%) | 0.179 |

HR: Heart Rate; MBP: Mean Blood Pressure; RR: Respiratory Rate; T: Temperature; SpO2: oxygen saturation; G: Glucose; PLT: Platelet; WBC: White Blood Cell; HCO3: Bicarbonate; BUN: Blood Urea Nitrogen; Cr: Creatinine; Na: Sodium; K: Potassium; INR: International Normalized Ratio; PT: Prothrombin Time; APTT: Activated Partial Thromboplastin Time; ALT: Alanine Aminotransferase; AST: Aspartate Aminotransferase; Hb: Hemoglobin; Fluid_3h_sum: Volume of intravenous infusion within 3h of ICU admission; Fluid_6h_sum: Volume of intravenous infusion within 6h of ICU admission; Fluid_24h_sum: Volume of intravenous infusion within 24h of ICU admission; AKI: Acute Kidney Injury; NEdose_24hmax: Maximum Norepinephrine Equivalent dose within 24 hours in ICU; Lac: Lactate; PO2: Arterial Oxygen Partial Pressure; PCO2: Arterial Carbon Dioxide Partial Pressure; P/F: Arterial Oxygen Partial Pressure / Fractional Inspired Oxygen; Charlson: Charlson comorbidity index; SAPS-II: Simplified Acute Physiology Score II; SAPS-III: Simplified Acute Physiology Score III; SIRS: Systemic Inflammatory Response Syndrome; SOFA: Sequential Organ Failure Assessment; CHF: Congestive heart failure; CVD: Cerebrovascular disease; CPD: Chronic pulmonary disease; RD: Rheumatic disease; CRD: Chronic renal disease. MV: Mechanical Ventilation.

**Appendix E. Variable selection using Lasso regression and Boruta algorithm.**

In Lasso regression analysis, a lambda value of 0.0008 was found to minimize the binomial deviance, indicating that 60 features were identified as the most significant predictors of the 90-day mortality rate.

The figure illustrates how variable coefficients change with different lambda values.


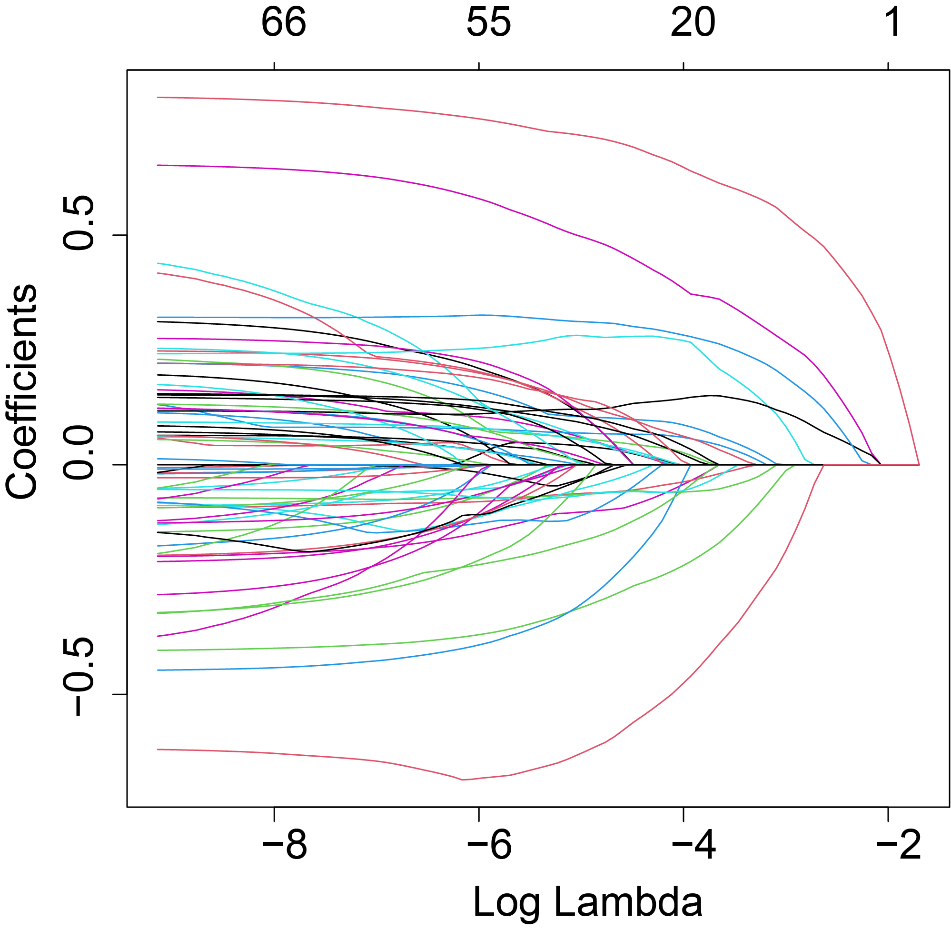


This figure illustrates the relationship between the LASSO regression regularization parameter (λ) and the regression coefficients of various variables. As λ increases, the regression coefficients of the variables gradually decrease. LASSO: Least Absolute Shrinkage and Selection Operator.


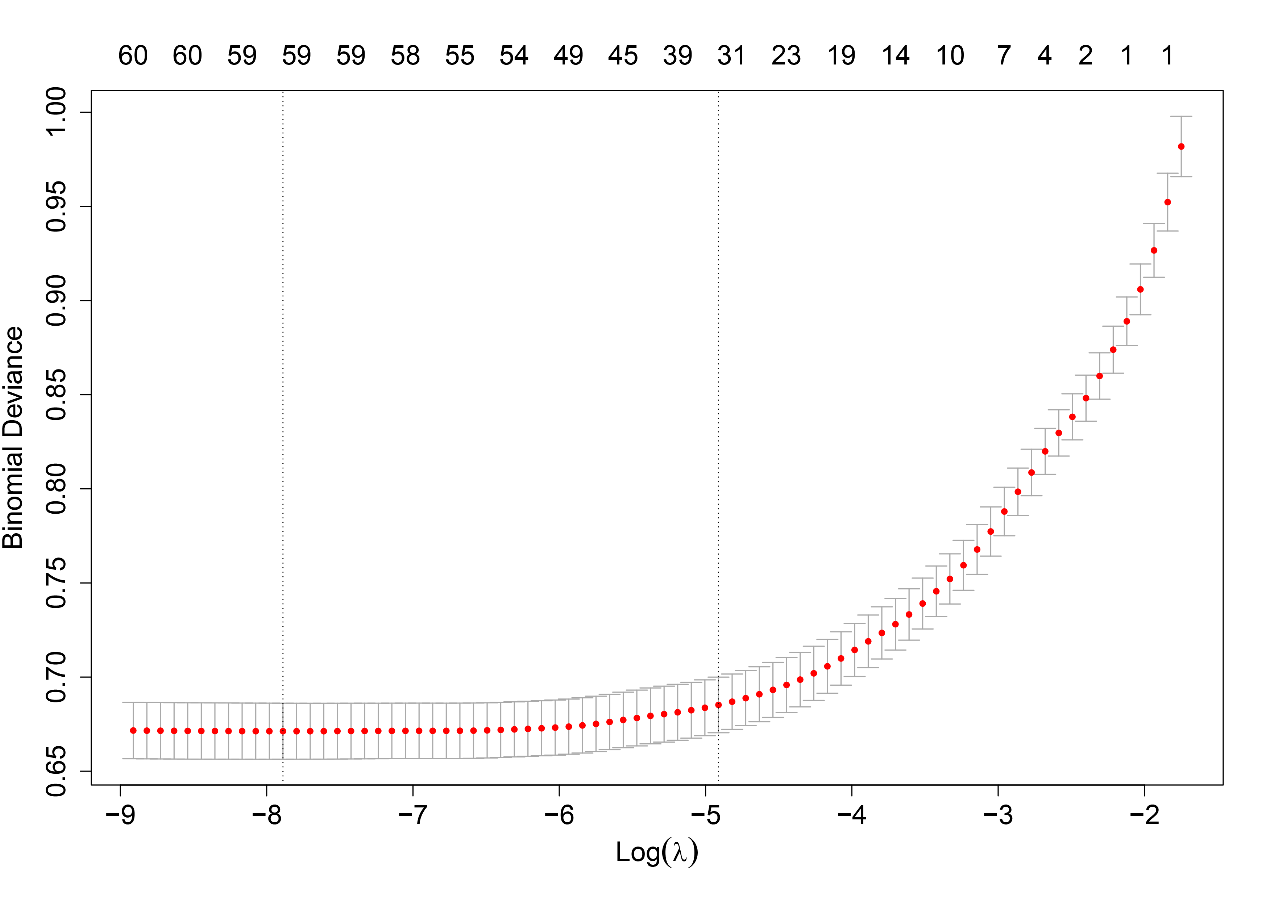
The figure illustrates the relationship between the LASSO regularization parameter lambda and the binomial deviance.

The relationship between the LASSO regression regularization parameter λ and binomial deviance shows that as λ increases, the binomial deviance also tends to increase. The minimum binomial deviance occurs at λ = 0.0008 (lambd.min), while at λ = 0.0064 (lambd.1se), the binomial deviance remains within one standard error.

Utilizing the Boruta algorithm for feature selection, we identified 54 variables that were confirmed to be important in predicting the 90-day mortality rate.

This figure shows the importance of the feature determined by the Boruta algorithm.


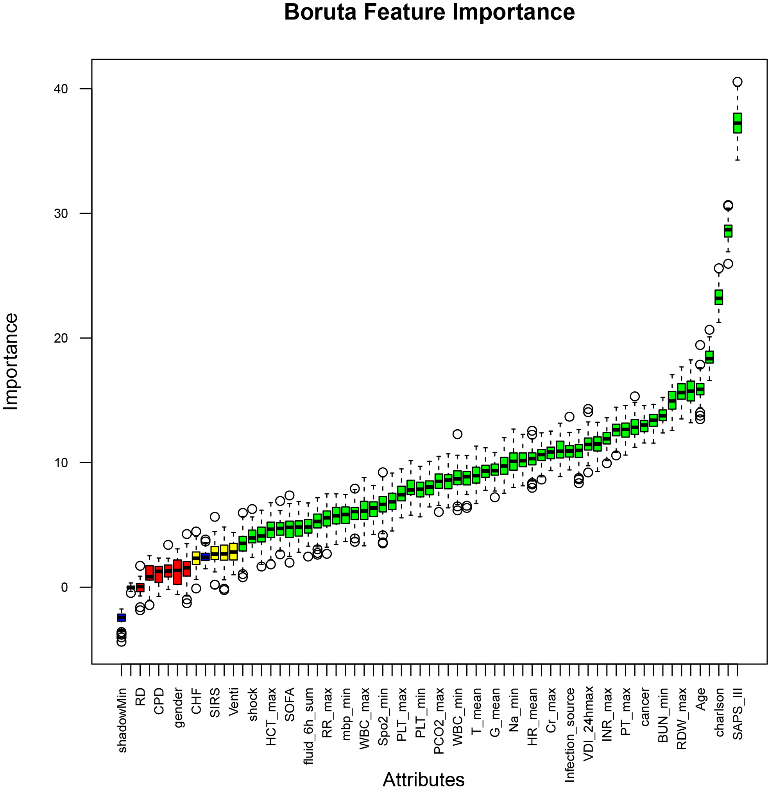


SAPS-III: Simplified Acute Physiology Score III; Charlson: Charlson comorbidity index; RDW: Red Cell Distribution Width; BUN: Blood Urea Nitrogen; PT: Prothrombin Time; INR: International Normalized Ratio; VDI_24hmax: Maximum Norepinephrine Equivalent dose within 24 hours in ICU; Cr: Creatinine; HR: Heart Rate; Na: Sodium; G: Glucose; T: Temperature; WBC: White Blood Cell; MBP: Mean Blood Pressure; RR: Respiratory Rate; SOFA: Sequential Organ Failure Assessment; HCT: Hematocrit; Venti: Mechanical Ventilation; SIRS: Systemic Inflammatory Response Syndrome; CHF: Congestive heart failure; CPD: Chronic pulmonary disease; RD: Rheumatic disease.


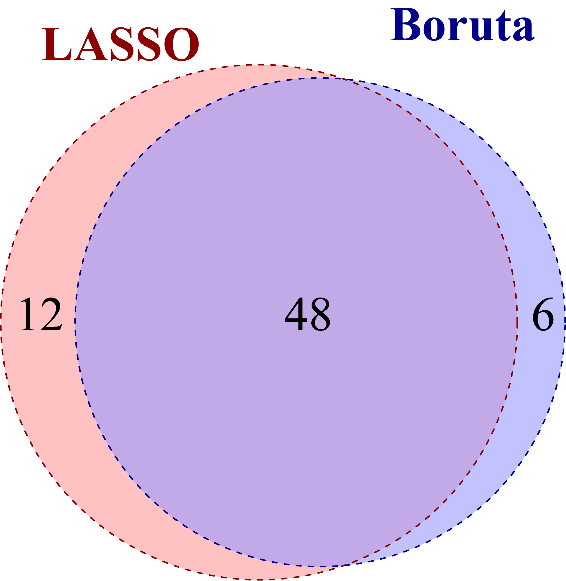
LASSO regression and the Boruta model selected 48 variables.


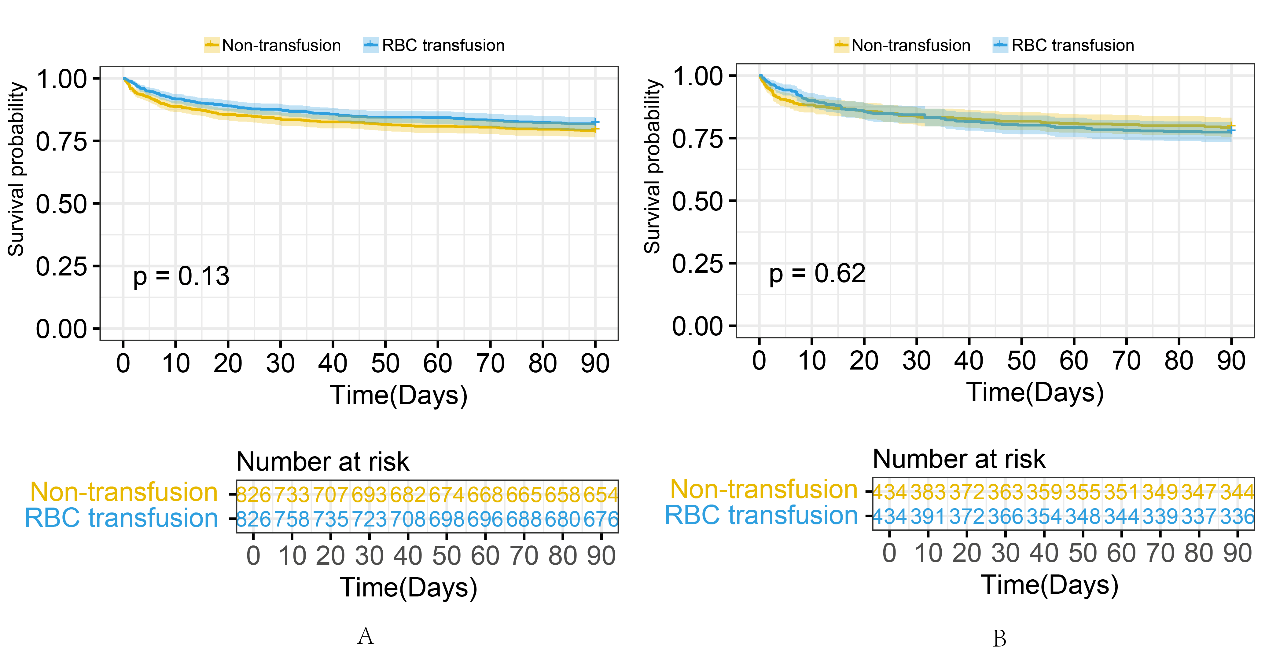
**Figure S1.** Kaplan-Meier curves for the RBC transfusion and non-transfusion groups in MIMIC-IV and MIMIC-III after propensity score matching.

1. Kaplan-Meier curves for the RBC transfusion and non-transfusion groups in MIMIC-IV; B Kaplan-Meier curves for the RBC transfusion and non-transfusion groups in MIMIC-III.

**Table S2:** Hemoglobin levels in the RBC transfusion and non-transfusion groups in the MIMIC-IV and MIMIC-III database after propensity score matching.

| Hb | MIMIC-IV | | | MIMIC-III | | |
| --- | --- | --- | --- | --- | --- | --- |
|  | Non-transfusion  N=826 | RBC transfusion  N=826 | p | Non-transfusion  N=434 | RBC transfusion  N=434 | p |
| ad_Hb(g/dL) | 8.80[8.00;9.90] | 8.90[8.10;10.00] | 0.194 | 8.80[7.90;9.80] | 8.70[8.20;9.80] | 0.437 |
| Hb_min(g/dL) | 7.80[7.40;8.30] | 7.80[7.33;8.20] | 0.019 | 8.00[7.60;8.50] | 8.00[7.60;8.40] | 0.176 |
| Hb_max(g/dL) | 10.20[9.30;11.10] | 10.30[9.60;11.10] | 0.009 | 10.25[9.50;11.08] | 10.50[9.70;11.28] | 0.010 |
| Hb_1d_min(g/dL) | 8.20[7.60;8.80] | 8.10[7.60;8.70] | 0.285 | 8.35[7.80;8.87] | 8.30[7.80;8.70] | 0.644 |
| Hb_2d_min(g/dL) | 8.60[8.00;9.30] | 8.70[8.00;9.50] | 0.348 | 9.00[8.40;9.60] | 8.90[8.40;9.90] | 0.569 |
| Hb_3d_min(g/dL) | 8.55[7.90;9.20] | 8.90[8.20;9.50] | <0.001 | 8.80[8.30;9.60] | 9.20[8.60;9.80] | <0.001 |
| Hb_4d_min(g/dL) | 8.60[8.00;9.20] | 8.90[8.30;9.60] | <0.001 | 9.00[8.40;9.77] | 9.30[8.70;10.00] | <0.001 |
| Hb_5d_min(g/dL) | 8.70[8.10;9.40] | 9.10[8.50;9.70] | <0.001 | 9.10[8.50;9.80] | 9.40[8.73;10.00] | <0.001 |
| Hb_1d_max(g/dL) | 10.10[9.10;11.00] | 10.00[9.10;10.90] | 0.599 | 10.00[9.30;10.98] | 10.00[9.10;10.90] | 0.673 |
| Hb_2d_max(g/dL) | 8.70[8.20;9.40] | 9.00[8.40;9.70] | <0.001 | 9.00[8.40;9.70] | 9.20[8.60;10.10] | 0.009 |
| Hb_3d_max(g/dL) | 8.70[8.10;9.30] | 9.00[8.40;9.70] | <0.001 | 9.00[8.40;9.70] | 9.35[8.70;9.90] | <0.001 |
| Hb_4d_max(g/dL) | 8.70[8.20;9.30] | 9.10[8.50;9.70] | <0.001 | 9.05[8.50;9.70] | 9.50[8.90;10.08] | <0.001 |
| Hb_5d_max(g/dL) | 8.80[8.30;9.40] | 9.10[8.50;9.90] | <0.001 | 9.05[8.50;9.80] | 9.40[8.70;10.10] | <0.001 |

ad_Hb: Hemoglobin at admission ICU; Hb_min: Minimum hemoglobin levels during the ICU stay; Hb_max: Maximum hemoglobin levels during the ICU stay; Hb_1d_min: Minimum hemoglobin level on the first day of ICU admission; Hb_2d_min: Minimum hemoglobin level on the secondary day of ICU admission; Hb_3d_min: Minimum hemoglobin level on the third day of ICU admission; Hb_4d_min: Minimum hemoglobin level on the fourth day of ICU admission; Hb_5d_min: Minimum hemoglobin level on the fifth day of ICU admission; Hb_1d_max: Maximum hemoglobin level on the first day of ICU admission; Hb_2d_max: Maximum hemoglobin level on the secondary day of ICU admission; Hb_3d_max: Maximum hemoglobin level on the third day of ICU admission; Hb_4d_max: Maximum hemoglobin level on the fourth day of ICU admission; Hb_5d_max Maximum hemoglobin level on the fifth day of ICU admission.

**Figure S2**. Ridgeline plot of hemoglobin levels across different time frames.
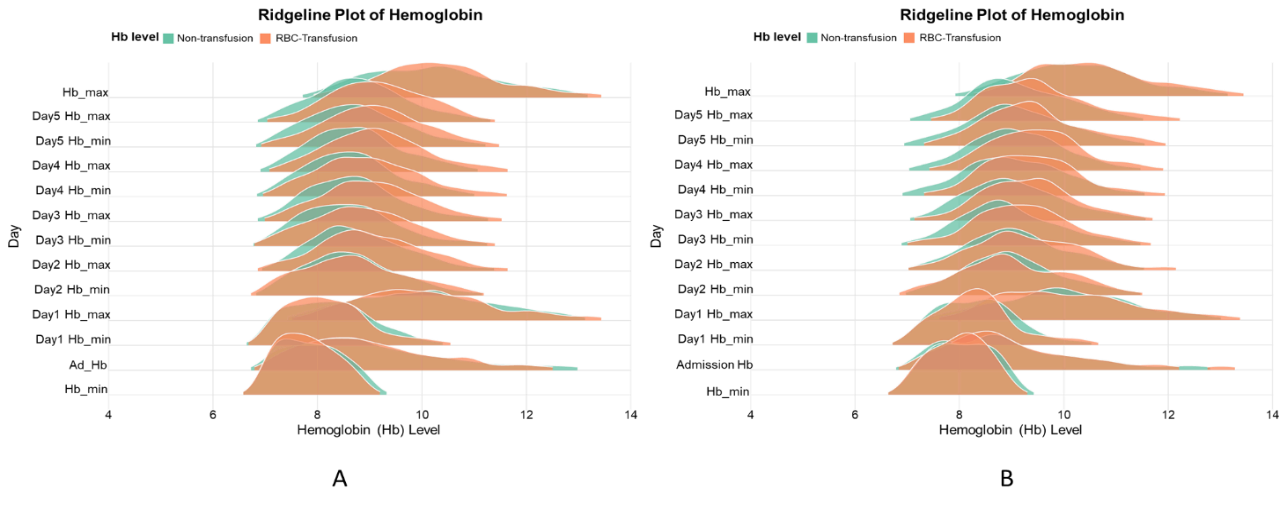


**A.** Distribution of hemoglobin levels in RBC transfusion and non-transfusion groups after propensity score matching in MIMIC-IV. **B.** Distribution of hemoglobin levels in RBC transfusion and non-transfusion groups after propensity score matching in MIMIC-III. ad_Hb: Hemoglobin at admission ICU; Hb_min: Minimum hemoglobin levels during the ICU stay; Hb_max: Maximum hemoglobin levels during the ICU stay; Hb_1d_min: Minimum hemoglobin level on the first day of ICU admission; Hb_2d_min: Minimum hemoglobin level on the secondary day of ICU admission; Hb_3d_min: Minimum hemoglobin level on the third day of ICU admission; Hb_4d_min: Minimum hemoglobin level on the fourth day of ICU admission; Hb_5d_min: Minimum hemoglobin level on the fifth day of ICU admission; Hb_1d_max: Maximum hemoglobin level on the first day of ICU admission; Hb_2d_max: Maximum hemoglobin level on the secondary day of ICU admission; Hb_3d_max: Maximum hemoglobin level on the third day of ICU admission; Hb_4d_max: Maximum hemoglobin level on the fourth day of ICU admission; Hb_5d_max Maximum hemoglobin level on the fifth day of ICU admission.

**Figure S3.** Hemoglobin trajectory classification plot.
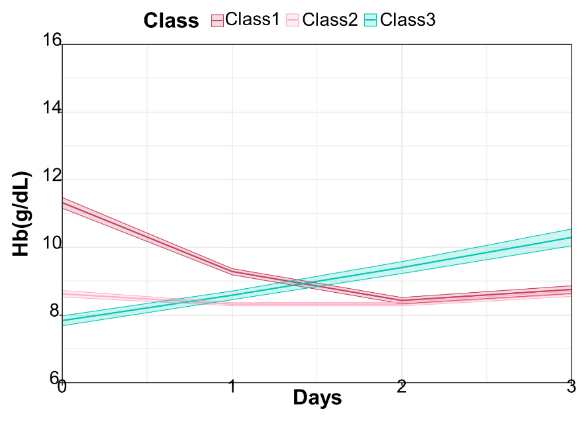


Hb hemoglobin; Class1: Decreasing class, Class2: Stable class, Class3: Increasing class.

**Figure S4.** Individual hemoglobin trajectories in the MIMIC-IV and MIMIC-III database after propensity score matching.


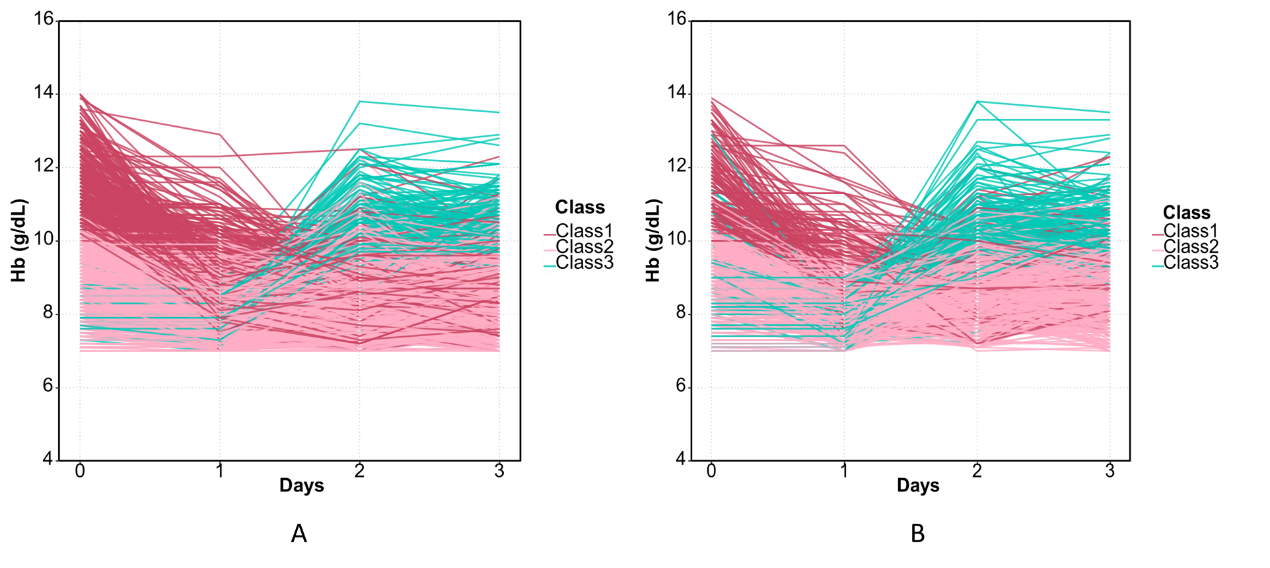


A. Hemoglobin Trajectories in MIMIC-IV Patients. B Hemoglobin Trajectories in MIMIC-III Patients. Hb hemoglobin; Class1: Decreasing class, Class2: Stable class, Class3: Increasing class.

**Figure S5.** Targeting Operator Characteristic (TOC) curve for evaluating the MIMIC−III validation cohort.


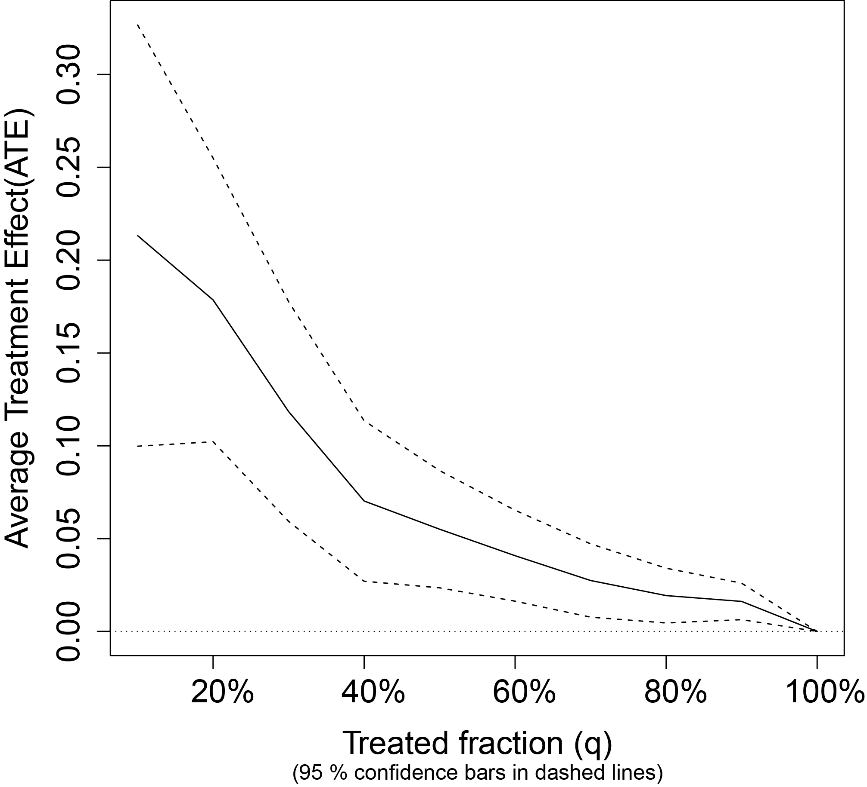


The TOC curve on the MIMIC-III data showed that the average treatment effect decreased as the treatment proportion increased.

**Figure S6.** Individual treatment effects (ITEs) on 90-day survival rates for patient subgroups across various quantiles.


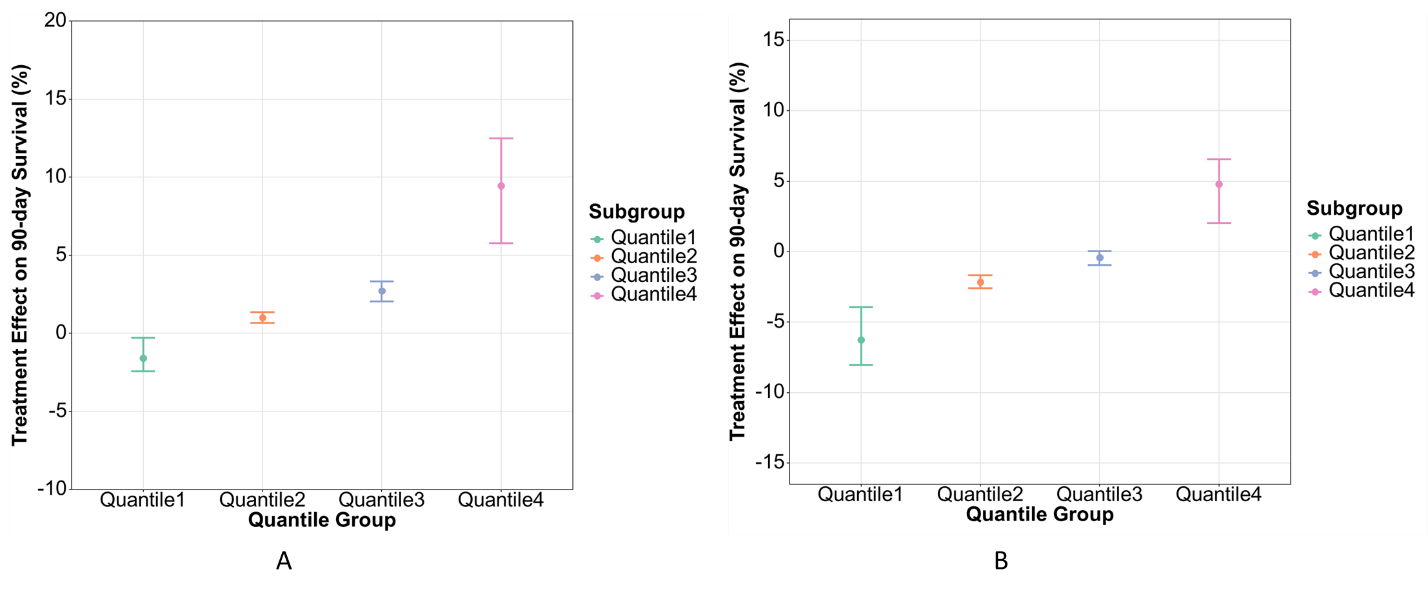


A. ITE for patient subgroups across different quantiles in the MIMIC-IV derivation cohort; B. ITE for patient subgroups across different quantiles in the MIMIC-III derivation cohort. ITE: Individual treatment effects.

**Figure S7.** The Kaplan-Meier curves for patients in Quantiles 1 to 4 subgroups of the MIMIC-IV validation cohort.
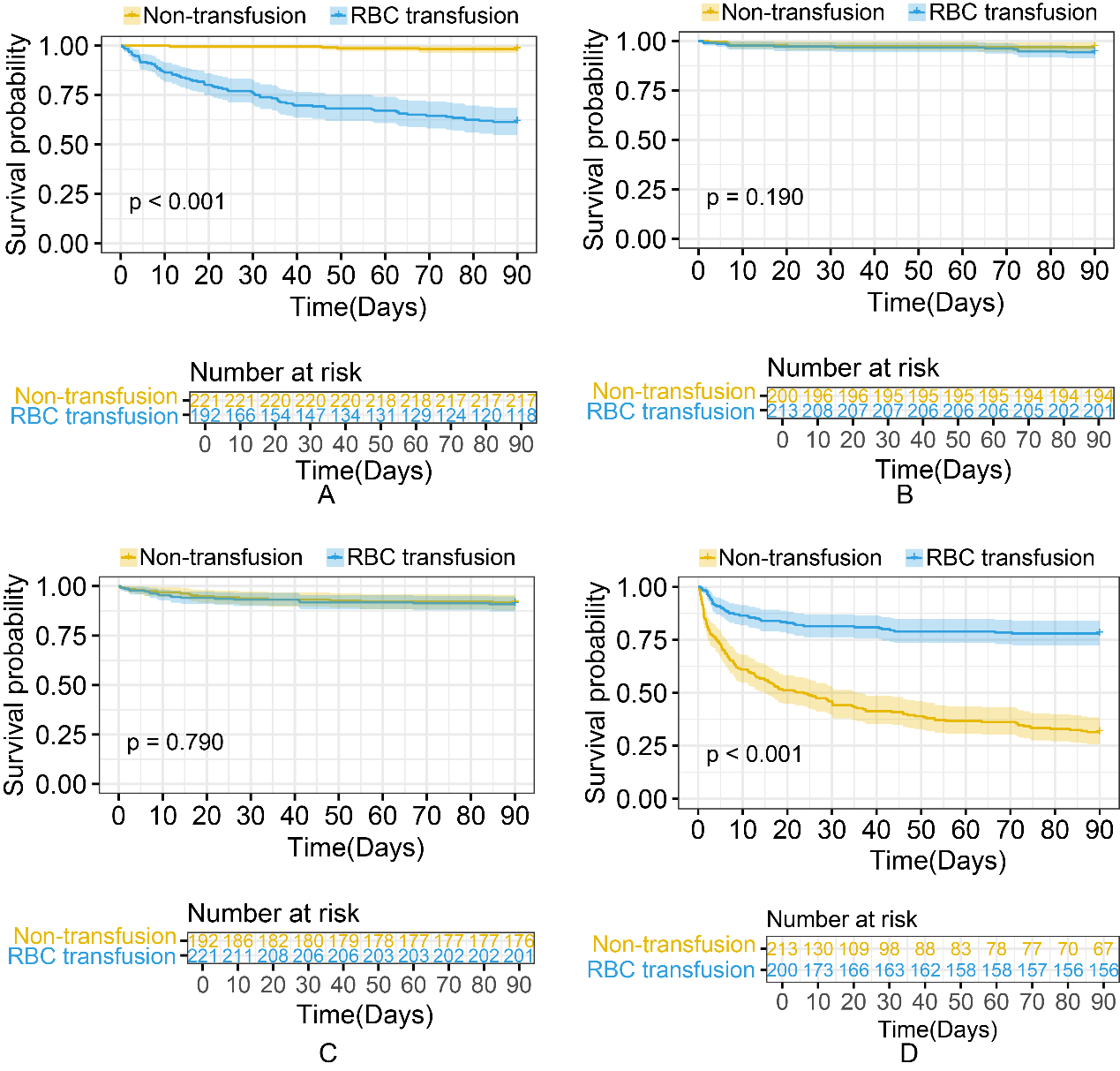


**A.**Quantile1 subgroup in MIMIC-III validation cohort; **B.**Quantile2 subgroup in MIMIC-III validation cohort; **C.**Quantile3 subgroup in MIMIC-III validation cohort; **D.**Quantile4 subgroup in MIMIC-III validation cohort. RBC Red Blood Cell.

**Table S3.** Differences in baseline characteristics among the different quantile subgroups.

| Factors | Quantile 1 | Quantile 2 | | Quantile 3 | | Quantile 4 | | p | |  |
| --- | --- | --- | --- | --- | --- | --- | --- | --- | --- | --- |
| MIMIC-IV | | | | | | | | | |  |
| SAPS_II | 35.00 [29.00;42.00] | 34.00 [28.00;40.00] | | 37.00 [31.00;45.00] | | 53.00 [42.00;62.00] | | <0.001 | |  |
| WBC_min | 7.10 [5.10;9.70] | 8.30 [6.40;10.60] | | 10.00 [7.80;12.50] | | 12.20 [7.40;17.30] | | <0.001 | |  |
| BUN_max | 19.00 [14.00;30.00] | 16.000 [13.00;22.00] | | 20.00 [14.00;27.00] | | 39.00 [26.00;57.00] | | <0.001 | |  |
| Na_min | 136.00 [134.00;139.00] | 137.00 [135.00;139.00] | | 137.00 [135.00;139.00] | | 136.00 [130.00;139.00] | | <0.001 | |  |
| HR_mean | 92.44 [83.76;99.81] | 81.13 [75.94;89.00] | | 79.79 [74.00;88.16] | | 82.35 [71.92;97.00] | | <0.001 | |  |
| PLT_min | 108.00 [72.00;151.00] | 128.00 [103.00;156.00] | | 151.00 [111.00;205.00] | | 171.00 [107.00;264.00] | | <0.001 | |  |
| RDW_min | 15.40 [14.10;17.30] | 13.70 [13.00;14.80] | | 14.10 [13.40;15.30] | | 15.80 [14.40;17.70] | | <0.001 | |  |
| Age | 64.54 [55.18;75.22] | 69.33 [61.14;75.59] | | 71.80 [61.01;81.68] | | 74.51 [62.35;84.60] | | <0.001 | |  |
| RR_max | 27.00 [24.00;31.00] | 25.00 [23.00;28.00] | | 26.00 [23.00;29.00] | | 28.00 [24.00;34.00] | | <0.001 | |  |
| HCO_3__min | 22.00 [20.00;24.00] | 22.00 [21.00;24.00] | | 22.00 [20.00;24.00] | | 19.00 [15.00;22.00] | | <0.001 | |  |
| MIMIC-III | | | | | | | | | |  |
| SAPS_II | 36.00 [29.00;42.00] | | 35.00 [29.00;42.00] | | 38.00 [31.00;49.00] | | 50.00 [36.00;60.00] | | <0.001 | |
| WBC_min | 6.60 [4.50;9.10] | | 8.70 [6.70;11.50] | | 9.80 [7.70;12.70] | | 10.80 [7.20;15.80] | | <0.001 | |
| BUN_max | 18.00 [15.00;31.00] | | 17.00 [14.00;25.00] | | 21.00 [15.00;31.00] | | 29.00 [17.00;46.00] | | <0.001 | |
| Na_min | 135.00 [133.00;138.00] | | 135.00 [134.00;137.000] | | 135.00 [133.00;137.00] | | 135.00 [133.00;138.00] | | 0.650 | |
| HR_mean | 85.50 [80.11;93.04] | | 82.43 [76.03;89.28] | | 87.94 [81.18;95.58] | | 99.38 [83.46;107.50] | | <0.001 | |
| PLT_min | 94.00 [67.00;131.00] | | 141.00 [115.00;190.00] | | 157.00 [125.00;205.00] | | 178.00 [114.00;368.00] | | <0.001 | |
| RDW_min | 15.20 [13.80;17.70] | | 14.20 [13.40;15.20] | | 14.30 [13.50;15.60] | | 15.80 [14.70;16.90] | | <0.001 | |
| Age | 71.33 [54.30;80.35] | | 73.97 [60.83;80.87] | | 65.59 [56.80;74.73] | | 69.13 [60.79;79.27] | | <0.001 | |
| RR_max | 26.00 [22.00;31.00] | | 25.00 [23.00;30.00] | | 27.00 [24.00;30.00] | | 28.00 [25.00;33.00] | | <0.001 | |
| HCO_3__min | 24.00 [21.00;26.00] | | 22.00 [21.00;24.00] | | 22.00 [20.00;24.00] | | 20.00 [17.00;24.00] | | <0.001 | |

SAPS-II: Simplified Acute Physiology Score II; WBC_min: Minimum white blood cell count within 24 hours in ICU; BUN_max: Maximum blood urea nitrogen within 24 hours in ICU; Na_min: Minimum sodium within 24 hours in ICU; HR_mean: Mean heart rate within 24 hours in ICU; PLT_min: Minimum platelet count within 24 hours in ICU; RDW_max: Maximum red cell distribution width within the first 24 hours of ICU admission; RR_max: Maximum respiratory rate within 24 hours in ICU; HCO3_min: Minimum bicarbonate within 24 hours in ICU.

Appendix E. R code for statistical analysis.

**# Load the required R packages**

| R packages | Introduce |
| --- | --- |
| glmnet (4.1-9) | Fit a LASSO regression model |
| Boruta(8.0.0) | Feature selection with the Boruta algorithm |
| survival(3.7-0) | Survival analysis |
| survminer(0.5.0) | Drawing Survival Curves using 'ggplot2' |
| MatchIt(4.7.2) | Perform propensity score matching analysis |
| lcmm(2.2.1) | Estimation of extended mixed models using latent classes and latent processes |
| LCTMtools(0.1.3) | Latent Class Trajectory Models: Tools for checking adequacy |
| grf(2.4.0) | Generalized Random Forests |

**# Build model with Lasso regression**

lasso_model <- glmnet(xlasso, # Predictor matrix

ylasso, # Response variable matrix

family = "binomial", # Binomial outcome (binary classification)

alpha = 1) # L1 regularization (Lasso for variable selection)

print(lasso_model)

# Cross-validation to select the optimal lambda

cv_model <- cv.glmnet(xlasso, ylasso, family = "binomial", alpha = 1, nfolds = 10)

lambda_min <- cv_model$lambda.min

lambda_min

# Build the final Lasso regression model

final_lasso_model <- coef(lasso_model,s=lambda_min)

final_lasso_model

lasso_result <- exp(final_lasso_model)

lasso_result <- as.matrix(lasso_result) # Convert to matrix

lasso_result <- data.frame(lasso_result) # Convert to data frame

lasso_result$Variable <- row.names(lasso_result)# Add variable names as a column

lasso_result <- lasso_result[-1, ] # Remove the intercept row

lasso_result <- lasso_result[lasso_result$s1 != 1, 2] # Filter out values where s1 is not 1 (modify as needed)

lasso_result

**# Feature selection using Boruta**

boruta_model <- Boruta(Death_90 ~ ., data = data_boruta,

doTrace = 2, # Show detailed output

maxRuns = 500, # Maximum number of iterations

pValue = 0.01, # Significance level

holdHistory = TRUE) # Save history

# Print the Boruta model result

print(boruta_model)

# Get the final confirmed important features

boruta_vars <- getSelectedAttributes(boruta_model, withTentative = FALSE)

print(boruta_vars)

**# Intersection of features selected by Lasso and Boruta**

selected_var <- intersect(lasso_result, boruta_vars)

selected_var

**Selected features**

“SOFA, weight, HR_max, HR_mean, mbp_min, RR_max,RR_mean, T_min, T_max, Spo2_min, Spo2_mean, G_min, G_max, G_mean, PLT_max, WBC_min,

WBC_max, HCT_min, HCO3_min, BUN_min, BUN_max, Cr_max, Cr_min, Na_min, Na_max, K_max, PT_max, APTT_max, NEdose_24hmax, Lac_max, PH_min, PO2_min, PCO2_max, CRD, cancer, charlson, SAPS_II, SAPS_III, RDW_min, Hb_1d_min, Hb_1d_max, fluid_3h_sum, fluid_6h_sum, shock, Age, Infection_source, type_ICU"

**# Build a propensity score matching model**

matchit_model <- matchit(RBC_trans ~ SOFA + weight + HR_max +

HR_mean + mbp_min + RR_max + RR_mean +

T_min + T_max + Spo2_min + Spo2_mean +

G_min + G_max + G_mean + PLT_max + WBC_min +

WBC_max + HCT_min + HCO3_min + BUN_min +

BUN_max + Cr_max + Cr_min + Na_min + Na_max +

K_max + PT_max + APTT_max + NEdose_24hmax +

Lac_max + PH_min + PO2_min + PCO2_max + CRD +

cancer + charlson + SAPS_II + SAPS_III + RDW_min +

Hb_1d_min + Hb_1d_max + fluid_3h_sum +

fluid_6h_sum + shock + Age + Infection_source + type_ICU,

data = data_MIMIC_IV,

distance = "logit", # Logistic distance for matching

method = "nearest", # Nearest neighbor matching

ratio = 1, # 1:1 matching ratio

caliper = 0.1, # Caliper width = 0.1

replace = FALSE) # Matching without replacement

summary(matchit_model)

**# Create survival object and fit MIMIC-IV Kaplan–Meier survival curves**

data_MIMIC_IV$Surv90 <- as.integer(data_MIMIC_IV$Death_90) - 1 # 0 = survived, 1 = death within 90 days

table(data_MIMIC_IV$Surv90)

colnames(data_MIMIC_IV)

# Fit Kaplan–Meier survival curves by transfusion status in the matched cohort

PSM_survive <- survfit(Surv(surtime, Surv90) ~ RBC_trans, data = data_MIMIC_IV)

# Visualize survival curves with risk tables and confidence intervals

PSM_ggsurv <- ggsurvplot(

PSM_survive,

data = data_MIMIC_IV,

conf.int = TRUE,

risk.table = TRUE,

risk.table.col = "strata",

pval = TRUE,

palette = c("#E7B800", "#2E9FDF"),

ggtheme = theme_bw(base_size = 21),

xlim = c(0, 90),

break.time.by = 10,

font.x = c(21, "plain", "black"), # X-axis label font

font.y = c(21, "plain", "black"), # Y-axis label font

font.tickslab = c(21, "plain", "black"), # Axis tick font size

tables.height = 0.3, # Height of risk table

risk.table.y.text = TRUE,

risk.table.fontsize = 6,

tables.theme = theme(axis.text = element_text(size = 21))

)

PSM_ggsurv

**# Build longitudinal trajectory models for Hb using latent class mixed models**

library(lcmm)

library(LCTMtools)

# Select and preprocess Hb variables from hospital days 0–3

table(Hbdata_match$Hb_day)

Hbdata_match <- Hbdata_match %>%

filter(Hb_day %in% c("Admission Hb","Day1 Hb_min","Day2 Hb_min","Day3 Hb_min")) %>%

mutate(day = case_when(

Hb_day == "Admission Hb" ~ 0,

Hb_day == "Day1 Hb_min" ~ 1,

Hb_day == "Day2 Hb_min" ~ 2,

Hb_day == "Day3 Hb_min" ~ 3

))

# Fit initial single-class (ng=1) model

start_model <- hlme(

fixed = Hb ~ day,

random = ~ day,

subject = "subject_id",

ng = 1,

data = Hbdata_match

)

lin <- c(start_model$ng, start_model$BIC)

# Fit models with 2–5 latent classes and compare BIC

for (i in 2:5) {

mi <- hlme(

fixed = Hb ~ day,

random = ~ day,

mixture = ~ day,

ng = i,

nwg = FALSE,

subject = "subject_id",

data = Hbdata_match,

B = start_model

)

lin <- rbind(lin, c(i, mi$BIC))

}

print(lin)

# Three-class model (lowest BIC)

m3 <- hlme(

fixed = Hb ~ day,

random = ~ day,

mixture = ~ day,

ng = 3,

nwg = FALSE,

subject = "subject_id",

data = Hbdata_match,

B = start_model

)

summary(m3)

m3$pprob

# Add quadratic effect of day

m1a <- hlme(

fixed = Hb ~ day + I(day^2),

random = ~ day,

ng = 1,

nwg = FALSE,

subject = "subject_id",

data = Hbdata_match

)

m3a <- hlme(

fixed = Hb ~ day + I(day^2),

random = ~ day,

mixture = ~ day,

ng = 3,

nwg = FALSE,

subject = "subject_id",

data = Hbdata_match,

B = m1a

)

summary(m3a)

# Add quadratic effect in both fixed and random effects

m1b <- hlme(

fixed = Hb ~ 1 + day + I(day^2),

random = ~ day + I(day^2),

ng = 1,

nwg = FALSE,

subject = "subject_id",

data = Hbdata_match

)

m3b <- hlme(

fixed = Hb ~ 1 + day + I(day^2),

random = ~ day + I(day^2),

mixture = ~ day,

ng = 3,

nwg = TRUE,

subject = "subject_id",

data = Hbdata_match,

B = m1b

)

summary(m3b)

# Add quadratic and intercept in mixture/random effects

m1c <- hlme(

fixed = Hb ~ 1 + day + I(day^2),

random = ~ 1 + day + I(day^2),

ng = 1,

nwg = FALSE,

subject = "subject_id",

data = Hbdata_match

)

m3c <- hlme(

fixed = Hb ~ 1 + day + I(day^2),

random = ~ 1 + day + I(day^2),

mixture = ~ 1 + day + I(day^2),

ng = 3,

nwg = TRUE,

subject = "subject_id",

data = Hbdata_match,

B = m1c

)

summary(m3c)

# Allow for heteroscedastic variances

m1d <- hlme(

fixed = Hb ~ 1 + day + I(day^2),

random = ~ 1 + day + I(day^2),

ng = 1,

nwg = FALSE,

idiag = TRUE,

subject = "subject_id",

data = Hbdata_match

)

m3d <- hlme(

fixed = Hb ~ 1 + day + I(day^2),

random = ~ 1 + day + I(day^2),

mixture = ~ 1 + day + I(day^2),

ng = 3,

nwg = FALSE,

idiag = TRUE,

subject = "subject_id",

data = Hbdata_match,

B = m1d

)

# Model comparison by BIC

m3a$BIC; m3b$BIC; m3c$BIC; m3d$BIC

summary(m3a)

summary(m3b)

summary(m3c)

summary(m3d)

m3d$pprob

table(m3d$pprob$class)

# Trajectory visualization using LCTMtools

LCTMtoolkit(m3a)

LCTMtoolkit(m3b)

LCTMtoolkit(m3c)

LCTMtoolkit(m3d)

# Choose model m3d as the best fit

# Predict and plot trajectory classes

Hb_newdata <- Hbdata_match[sample(nrow(Hbdata_match), 200), ] %>%

select(day, subject_id)

plotpred <- predictY(m3d,

Hb_newdata,

var.time = "day",

draws = TRUE,

level = FALSE) # Predict Y values

data_Hb_pre <- data.frame(plotpred$pred, plotpred$times)

colnames(data_Hb_pre) <- c(

"Mean_class1","Mean_class2","Mean_class3",

"Lower_class1","Lower_class2","Lower_class3",

"High_class1","High_class2","High_class3","day"

)

data_Hb_pre$id <- 1:200

# Reshape for plotting

library(tidyr)

data_Hb_pre <- pivot_longer(

data_Hb_pre, cols = contains(c("Mean")), names_to = "Mean_class", values_to = "Mean"

)

data_Hb_pre <- pivot_longer(

data_Hb_pre, cols = contains(c("Lower")), names_to = "Lower_class", values_to = "Lower"

)

data_Hb_pre <- pivot_longer(

data_Hb_pre, cols = contains(c("High")), names_to = "High_class", values_to = "High"

)

data_Hb_pre$Mean_class <- factor(data_Hb_pre$Mean_class,

levels = c("Mean_class1","Mean_class2","Mean_class3"),

labels = c("Class1","Class2","Class3"))

data_Hb_pre$Lower_class <- factor(data_Hb_pre$Lower_class,

levels = c("Lower_class1","Lower_class2","Lower_class3"),

labels = c("Class1","Class2","Class3"))

data_Hb_pre$High_class <- factor(data_Hb_pre$High_class,

levels = c("High_class1","High_class2","High_class3"),

labels = c("Class1","Class2","Class3"))

data_Hb_pre <- arrange(data_Hb_pre, id, day, Mean_class, Lower_class, High_class)

data_Hb_pre <- filter(data_Hb_pre, Mean_class == Lower_class & Lower_class == High_class)

# Hb trajectory plot—mean and confidence intervals by class

colors <- c("Class1" = "#ca4362", "Class2" = "#ffaec6", "Class3" = "#00c9b6")

m3d_pre_fig <- ggplot(data_Hb_pre, aes(x = day, y = Mean, color = Mean_class)) +

geom_line(linewidth = 1) +

geom_ribbon(aes(ymin = Lower, ymax = High, fill = Mean_class), alpha = 0.2) +

scale_color_manual(values = colors) +

scale_fill_manual(values = colors) +

labs(x = "Day", y = "Hb", color = "Class", fill = "Class") +

theme_bw(base_size = 15) +

scale_x_continuous(expand = c(0, 0), limits = c(0, 3)) +

scale_y_continuous(expand = c(0, 0), limits = c(6, 16)) +

theme(

plot.title = element_text(hjust = 0.5, face = "bold"),

axis.text = element_text(size = 12),

axis.title = element_text(size = 14, face = "bold"),

legend.position = "top",

legend.title = element_text(face = "bold"),

legend.key = element_blank()

)

# Export plot as .pptx

graph2office(x = m3d_pre_fig, file = "Hb_trajectory.pptx", type = "PPT", width = 10, height = 7.5)

print(m3d_pre_fig)

# Assign class to matched data

Hb_class <- m3d$pprob %>% select(subject_id, class)

dataC_matched <- left_join(dataC_matched, Hb_class, by = "subject_id")

table(dataC_matched$class)

# Plot trajectories for individual patients, colored by class

Hbdata_match <- left_join(Hbdata_match, Hb_class, by = "subject_id")

Hbdata_match$class <- factor(Hbdata_match$class, levels = c(1,2,3), labels = c("Class1","Class2","Class3"))

m3d_fig <- ggplot(Hbdata_match, aes(x = day, y = Hb, color = class)) +

geom_line(aes(group = subject_id), alpha = 0.8, linewidth = 1) +

scale_color_manual(values = colors) +

xlab("Day") +

ylab("Hb (g/dL)") +

labs(color = "Class Assignment") +

scale_y_continuous(expand = c(0,0), limits = c(4, 16)) +

theme_bw(base_size = 16) +

theme(

axis.title = element_text(size = 14, face = "bold"),

axis.text = element_text(size = 18),

legend.title = element_text(size = 14, face = "bold"),

legend.text = element_text(size = 14),

panel.border = element_rect(color = "black", size = 1),

panel.grid.major = element_line(size = 0.5, linetype = "dotted", color = "grey80"),

panel.grid.minor = element_blank(),

legend.position = "right"

) +

guides(color = guide_legend(override.aes = list(size = 4)))

print(m3d_fig)

graph2office(x = m3d_fig, file = "MIMIC-IV_Hb_trajectory.pptx", type = "PPT", width = 10, height = 7.5)

**# Build a Causal Forest model**

W <- data_forest$RBC_trans # Treatment variable (binary: transfusion)

Y <- data_forest$Surv90 # Outcome variable (binary: 90-day survival)

colnames(data_forest)

X <- data_forest %>%

dplyr::select(-c("RBC_trans", "Surv90" ))

**# Fit the initial causal forest**

cf <- causal_forest(

X, Y, W,

num.trees = 5000,

honesty = TRUE,

seed = 999,

tune.parameters = "all",

tune.num.trees = 2000 # Number of trees used for tuning hyperparameters

)

# View variable importance ranking using the variable_importance() function

var_imp <- c(variable_importance(cf))

names(var_imp) <- colnames(X)

var_imp <- sort(var_imp, decreasing = TRUE)

pre_10_bvar <- names(var_imp)[1:10] # Extract the top 10 most important features

pre_10_bvar

**# Refit the causal forest using only the top 10 most important features**

X_b <- X %>% select(all_of(pre_10_bvar))

cf_b <- causal_forest(

X_b, Y, W,

num.trees = 5000,

sample.fraction = 0.5, # Fraction of sample used to build each tree

honesty = TRUE,

seed = 999,

tune.parameters = c(

"mtry", "alpha", "imbalance.penalty",

"honesty.prune.leaves", "min.node.size", "honesty.fraction"

),

tune.num.trees = 2000 # Number of trees for tuning

)

cf_b$tunable.params

test_calibration(cf_b) # Check calibration on the training set

# Check variable importance ranking using variable_importance() for the refined forest

varb_imp <- c(variable_importance(cf_b))

names(varb_imp) <- colnames(X_b)

varb_imp <- sort(varb_imp, decreasing = TRUE)

varb_imp

pre_10_bvar <- names(varb_imp)[1:10] # Extract the top 10 most important features

pre_10_bvar

**# Display variable importance ranking**

xbvars <- colnames(X_b)

imp_b <- sort(setNames(variable_importance(cf_b), xbvars))

barplot(imp_b, horiz = TRUE, las = 1, col = "orange")

**# Plot ITE (Individual Treatment Effect) curves for MIMIC-IV using a causal forest**

tau.hat <- predict(cf_b, X_b, estimate.variance = TRUE)

colnames(tau.hat)

tau.hat <- tau.hat %>%

arrange(predictions) %>%

mutate(sigma = sqrt(variance.estimates)) %>%

mutate(

High = predictions + 1.96 * sigma ,

Low = predictions - 1.96 * sigma

)

tau.hat$id <- seq(1, nrow(tau.hat), by = 1)

tau.hat <- tau.hat %>%

mutate(

predictions = predictions * 100,

High = High * 100,

Low = Low * 100

)

ITE_IV <- ggplot(tau.hat, aes(x = id, y = predictions)) +

# Add confidence ribbon

geom_ribbon(aes(ymin = Low, ymax = High), fill = "#D7BDE2", alpha = 0.9) +

# Add line for predicted ITEs

geom_line(color = "#845ec2", size = 1) +

labs(

x = "Individual Ranking of Treatment Effects",

y = "Treatment Effect on 90-day Survival (%)"

) +

theme_minimal(base_size = 21) +

theme(

plot.title = element_text(face = "bold", size = 18, hjust = 0.5),

plot.subtitle = element_text(size = 21, hjust = 0.5, face = "italic"),

axis.title = element_text(face = "bold", size = 21),

axis.text = element_text(size = 21),

plot.caption = element_text(hjust = 1, face = "italic", size = 10),

panel.grid.major = element_line(color = "gray"),

panel.grid.minor = element_blank()

)

ITE_IV

# Export the plot as a PowerPoint slide

graph2office(x = ITE_IV, file = "MIMIC-IV_ITE.pptx",

type = "PPT", width = 10, height = 7.5)

**# Estimate the average treatment effect (ATE) for the treated group**

ate_results <- average_treatment_effect(cf_b, target.sample = "treated")

# Print results

print(ate_results)

# Extract results

estimate <- ate_results[1] # Estimated ATE

std_error <- ate_results[2] # Standard error of the ATE

# Calculate t-statistic

t_stat <- estimate / std_error

# Calculate two-sided p-value

p_value <- 2 * pnorm(-abs(t_stat))

p_value

# Output results

cat("Average Treatment Effect estimate: ", estimate, "\n")

cat("Standard Error: ", std_error, "\n")

cat("t-statistic: ", t_stat, "\n")

cat("p-value: ", p_value, "\n")

# Check statistical significance at alpha = 0.05

if (p_value <= 0.05) {

cat("The treatment effect is statistically significant.\n")

} else {

cat("The treatment effect is not statistically significant.\n")

}

**# Predict individual treatment effects (ITE) and divide patients into quartiles**

tau.hatA <- predict(cf_b, X_b, estimate.variance = TRUE)

tau.hatA <- tau.hatA$predictions

ITE_data <- cbind(data_forest, tau.hatA)

ITE_data$subgroup <- cut(ITE_data$tau.hatA,

breaks = quantile(ITE_data$tau.hatA, probs = c(0, 0.25, 0.5, 0.75, 1)),

labels = c("sub_group1", "sub_group2", "sub_group3", "sub_group4"),

include.lowest = TRUE)

table(ITE_data$subgroup)

**## survival curve plotting by ITE quartile in MIMIC-IV**

# Quantile 1

PSM_survive_IV_1 <- survfit(Surv(surtime, Death_90) ~ RBC_trans,

data = ITE_data[ITE_data$subgroup=="sub_group1",])

PSM_ggsurv_IV_1 <- ggsurvplot(PSM_survive_IV_1,

data = ITE_data[ITE_data$subgroup=="sub_group1",],

conf.int = TRUE,

risk.table = TRUE, risk.table.col = "strata", pval = TRUE,

palette = c("#E7B800", "#2E9FDF"),

ggtheme = theme_bw(base_size = 24),

xlim = c(0, 90),

break.time.by = 10,

font.x = c(24, "plain", "black"),

font.y = c(24, "plain", "black"),

font.tickslab = c(24, "plain", "black"),

tables.height = 0.3,

risk.table.y.text = TRUE,

risk.table.fontsize = 6,

tables.theme = theme(axis.text = element_text(size = 21))

)

PSM_ggsurv_IV_1

pdf("MIMIC_iv_Quantile1.PDF")

PSM_ggsurv_IV_1

dev.off()

# Repeat for Quantile 2

PSM_survive_IV_2 <- survfit(Surv(surtime, Death_90) ~ RBC_trans,

data = ITE_data[ITE_data$subgroup=="sub_group2",])

PSM_ggsurv_IV_2 <- ggsurvplot(PSM_survive_IV_2,

data = ITE_data[ITE_data$subgroup=="sub_group2",],

conf.int = TRUE,

risk.table = TRUE, risk.table.col = "strata", pval = TRUE,

palette = c("#E7B800", "#2E9FDF"),

ggtheme = theme_bw(base_size = 24),

xlim = c(0, 90),

break.time.by = 10,

font.x = c(24, "plain", "black"),

font.y = c(24, "plain", "black"),

font.tickslab = c(24, "plain", "black"),

tables.height = 0.3,

risk.table.y.text = TRUE,

risk.table.fontsize = 6,

tables.theme = theme(axis.text = element_text(size = 21))

)

PSM_ggsurv_IV_2

pdf("MIMIC_iv_Quantile2.PDF")

PSM_ggsurv_IV_2

dev.off()

# Quantile 3

PSM_survive_IV_3 <- survfit(Surv(surtime, Death_90) ~ RBC_trans,

data = ITE_data[ITE_data$subgroup=="sub_group3",])

PSM_ggsurv_IV_3 <- ggsurvplot(PSM_survive_IV_3,

data = ITE_data[ITE_data$subgroup=="sub_group3",],

conf.int = TRUE,

risk.table = TRUE, risk.table.col = "strata", pval = TRUE,

palette = c("#E7B800", "#2E9FDF"),

ggtheme = theme_bw(base_size = 24),

xlim = c(0, 90),

break.time.by = 10,

font.x = c(24, "plain", "black"),

font.y = c(24, "plain", "black"),

font.tickslab = c(24, "plain", "black"),

tables.height = 0.3,

risk.table.y.text = TRUE,

risk.table.fontsize = 6,

tables.theme = theme(axis.text = element_text(size = 21))

)

PSM_ggsurv_IV_3

pdf("MIMIC_iv_Quantile3.PDF")

PSM_ggsurv_IV_3

dev.off()

# Quantile 4

PSM_survive_IV_4 <- survfit(Surv(surtime, Death_90) ~ RBC_trans,

data = ITE_data[ITE_data$subgroup=="sub_group4",])

PSM_ggsurv_IV_4 <- ggsurvplot(PSM_survive_IV_4,

data = ITE_data[ITE_data$subgroup=="sub_group4",],

conf.int = TRUE,

risk.table = TRUE, risk.table.col = "strata", pval = TRUE,

palette = c("#E7B800", "#2E9FDF"),

ggtheme = theme_bw(base_size = 21),

xlim = c(0, 90),

break.time.by = 10,

font.x = c(24, "plain", "black"),

font.y = c(24, "plain", "black"),

font.tickslab = c(24, "plain", "black"),

tables.height = 0.3,

risk.table.y.text = TRUE,

risk.table.fontsize = 6,

tables.theme = theme(axis.text = element_text(size = 21))

)

PSM_ggsurv_IV_4

pdf("MIMIC_iv_Quantile4.PDF")

PSM_ggsurv_IV_4

dev.off()

**######## Export Partial Dependence Plots for Each Variable #########**

colnames(X_b)

X_b <- X_b %>% dplyr::select(all_of(pre_10_bvar))

**# Build partial dependence function**

partial_dependence <- function(grid, cf_b, variable_index) {

X.new <- X_b

X.new[, variable_index] <- grid

tau.pred <- predict(cf_b, newdata = X.new)$predictions

mean(tau.pred)

}

colnames(X_b)

# 1. SAPS_II

colnames(X_b)

summary(X_b$SAPS_II)

variable_index <- 1

variable_name <- "SAPS_II"

grid <- seq(min(X_b[, variable_index]), max(X_b[, variable_index]), length.out = 100)

# Compute partial dependence

SAPS_II_pd_effects <- sapply(

grid,

partial_dependence,

cf = cf_b, variable_index = variable_index

)

# Plot

SAPS_II_pd_data <- data.frame(grid = grid, effect = SAPS_II_pd_effects)

SAPS_II_pd <- ggplot(SAPS_II_pd_data, aes(x = grid, y = effect)) +

geom_smooth(method = "loess", span = 0.2, color = "steelblue", size = 1.0) +

labs(x = variable_name, y = "Estimated Treatment Effect (CATE)") +

theme_minimal(base_size = 21)

SAPS_II_pd

# 2. WBC_min

colnames(X_b)

summary(X_b$WBC_min)

variable_index <- 2

variable_name <- "WBC_min"

grid <- seq(min(X_b[, variable_index]), max(X_b[, variable_index]), length.out = 100)

WBC_min_effects <- sapply(grid, partial_dependence, cf = cf_b, variable_index = variable_index)

WBC_min_data <- data.frame(grid = grid, effect = WBC_min_effects)

WBC_min_pd <- ggplot(WBC_min_data, aes(x = grid, y = effect)) +

scale_x_continuous(limits = c(0,19)) +

geom_smooth(method = "loess", span = 0.2, color = "steelblue", size = 1.0) +

labs(x = variable_name, y = "Estimated Treatment Effect (CATE)") +

theme_minimal(base_size = 21)

WBC_min_pd

# Continue similarly for all 10 variables...

# 3. BUN_max ...

# 4. Na_min ...

# 5. HR_mean ...

# 6. PLT_min ...

# 7. RDW_min ...

# 8. Age ...

# 9. RR_max ...

# 10. HCO3_min ...

# Combine plots using patchwork

library(patchwork)

colnames(X_b)

rm(combined_plot)

combined_plot <- (SAPS_II_pd | WBC_min_pd | BUN_max_pd | Na_min_pd | HR_mean_pd) /

(PLT_min_pd | RDW_min_pd | Age_pd | RR_max_pd | HCO3_min_pd)

combined_plot

# Export to Office (PPT)

graph2office(x = combined_plot, file = "combined_plot.pptx",

type = "PPT", width = 16, height = 8)

Here is your code fully translated to English (including comments and labels), with a little cleaned formatting for clarity:

**####### Plot the ITE curve for MIMIC-III######**

tau.hat_III <- predict(cf_b, X_III, estimate.variance = TRUE)

colnames(tau.hat_III)

tau.hat_III <- tau.hat_III %>%

arrange(predictions) %>%

mutate(

sigma = sqrt(variance.estimates),

High = predictions + 1.96 * sigma,

Low = predictions - 1.96 * sigma

)

tau.hat_III$id <- seq(1, nrow(tau.hat_III), by = 1)

tau.hat_III <- tau.hat_III %>%

mutate(

predictions = predictions * 100,

High = High * 100,

Low = Low * 100

)

MIMIC_III_ITE <- ggplot(tau.hat_III, aes(x = id, y = predictions)) +

# Draw confidence interval as a translucent ribbon

geom_ribbon(aes(ymin = Low, ymax = High), fill = "#D7BDE2", alpha = 0.5) +

# Draw main line (trend of predicted values)

geom_line(color = "#845ec2", size = 1) +

scale_y_continuous(limits = c(-30, 30), breaks = seq(-30, 30, by = 10)) +

# Add main title and axis labels

labs(

x = "Individual Ranking of Treatment Effects",

y = "Treatment Effect on 90-day Survival (%)"

) +

# Modify theme

theme_minimal(base_size = 21) +

theme(

plot.title = element_text(face = "bold", size = 21, hjust = 0.5), # Center and bold title

plot.subtitle = element_text(size = 21, hjust = 0.5, face = "italic"), # Subtitle style

axis.title = element_text(face = "bold", size = 21), # Bold axis labels

axis.text = element_text(size = 21), # Axis tick size

plot.caption = element_text(hjust = 1, face = "italic", size = 21), # Right align caption

panel.grid.major = element_line(color = "gray"), # Major gridline style

panel.grid.minor = element_blank() # Remove minor gridlines

)

MIMIC_III_ITE

# Export to PowerPoint

graph2office(

x = MIMIC_III_ITE,

file = "MIMIC-III_ITE.pptx",

type = "PPT", width = 10, height = 7.5

)

**########## Predict individual treatment effects and MIMIC-III variances########**

**Extract final model parameters**

sample.fraction <-cf_b$tunable.params$sample.fraction

mtry <- cf_b$tunable.params$mtry

min.node.size <-cf_b$tunable.params$min.node.size

honesty.fraction <- cf_b$tunable.params$honesty.fraction

honesty.prune.leaves <- cf_b$tunable.params$honesty.prune.leaves

alpha <- cf_b$tunable.params$alpha

imbalance.penalty <- cf_b$tunable.params$imbalance.penalty

eval.forest_III <- causal_forest(X_III, Y_III ,W_III , num.trees = 5000,

seed = 999,

min.node.size = min.node.size,

sample.fraction = sample.fraction,

mtry = mtry,

honesty.fraction =honesty.fraction,

honesty.prune.leaves = honesty.prune.leaves,

alpha = alpha,

imbalance.penalty = imbalance.penalty )

tau.hat.IIIA <- predict(cf_b, X_III)$predictions

rate.cate.III <- rank_average_treatment_effect(eval.forest_III,tau.hat.IIIA)

rate.cate.III

plot(rate.cate.III , main = "TOC evaluated on MIMIC-III estimated from MIMIC-IV",

ylab = "Average Treatment Effect(ATE)")

**# Kaplan-Meier Survival Curves for Different Quantile-based Subgroups in MIMIC-III**

ITE_data_III <- cbind(data_forest_III, tau.hat.IIIA)

# Divide into quartile subgroups based on ITE

ITE_data_III$subgroup <- cut(

ITE_data_III$tau.hat.III,

breaks = quantile(ITE_data_III$tau.hat.III, probs = c(0, 0.25, 0.5, 0.75, 1)),

labels = c("sub_group1", "sub_group2", "sub_group3", "sub_group4"),

include.lowest = TRUE

)

table(ITE_data_III$subgroup)

# Quantile 1

PSM_survive_III_1 <- survfit(Surv(surtime, Death_90) ~ RBC_trans,

data = ITE_data_III[ITE_data_III$subgroup=="sub_group1",])

PSM_ggsurv_III_1 <- ggsurvplot(PSM_survive_III_1,

data = ITE_data_III[ITE_data_III$subgroup=="sub_group1",],

conf.int = TRUE,

risk.table = TRUE, risk.table.col = "strata", pval = TRUE,

palette = c("#E7B800", "#2E9FDF"),

ggtheme = theme_bw(base_size = 21),

xlim = c(0, 90),

break.time.by = 10,

font.x = c(21, "plain", "black"), # x axis label font

font.y = c(21, "plain", "black"), # y axis label font

font.tickslab = c(21, "plain", "black"), # axis tick font

tables.height = 0.3,

risk.table.y.text = TRUE,

risk.table.fontsize = 6,

tables.theme = theme(axis.text = element_text(size = 21))

)

PSM_ggsurv_III_1

pdf("MIMIC_III_Quantile1.PDF")

PSM_ggsurv_III_1

dev.off()

# Quantile 2

PSM_survive_III_2 <- survfit(Surv(surtime, Death_90) ~ RBC_trans,

data = ITE_data_III[ITE_data_III$subgroup=="sub_group2",])

PSM_ggsurv_III_2 <- ggsurvplot(PSM_survive_III_2,

data = ITE_data_III[ITE_data_III$subgroup=="sub_group2",],

conf.int = TRUE,

risk.table = TRUE, risk.table.col = "strata", pval = TRUE,

palette = c("#E7B800", "#2E9FDF"),

ggtheme = theme_bw(base_size = 21),

xlim = c(0, 90),

break.time.by = 10,

font.x = c(21, "plain", "black"),

font.y = c(21, "plain", "black"),

font.tickslab = c(21, "plain", "black"),

tables.height = 0.3,

risk.table.y.text = TRUE,

risk.table.fontsize = 6,

tables.theme = theme(axis.text = element_text(size = 21))

)

PSM_ggsurv_III_2

pdf("MIMIC_III_Quantile2.PDF")

PSM_ggsurv_III_2

dev.off()

# Quantile 3

PSM_survive_III_3 <- survfit(Surv(surtime, Death_90) ~ RBC_trans,

data = ITE_data_III[ITE_data_III$subgroup=="sub_group3",])

PSM_ggsurv_III_3 <- ggsurvplot(PSM_survive_III_3,

data = ITE_data_III[ITE_data_III$subgroup=="sub_group3",],

conf.int = TRUE,

risk.table = TRUE, risk.table.col = "strata", pval = TRUE,

palette = c("#E7B800", "#2E9FDF"),

ggtheme = theme_bw(base_size = 21),

xlim = c(0, 90),

break.time.by = 10,

font.x = c(21, "plain", "black"),

font.y = c(21, "plain", "black"),

font.tickslab = c(21, "plain", "black"),

tables.height = 0.3,

risk.table.y.text = TRUE,

risk.table.fontsize = 6,

tables.theme = theme(axis.text = element_text(size = 21))

)

PSM_ggsurv_III_3

pdf("MIMIC_III_Quantile3.PDF")

PSM_ggsurv_III_3

dev.off()

# Quantile 4

PSM_survive_III_4 <- survfit(Surv(surtime, Death_90) ~ RBC_trans,

data = ITE_data_III[ITE_data_III$subgroup=="sub_group4",])

PSM_ggsurv_III_4 <- ggsurvplot(PSM_survive_III_4,

data = ITE_data_III[ITE_data_III$subgroup=="sub_group4",],

conf.int = TRUE,

risk.table = TRUE, risk.table.col = "strata", pval = TRUE,

palette = c("#E7B800", "#2E9FDF"),

ggtheme = theme_bw(base_size = 21),

xlim = c(0, 90),

break.time.by = 10,

font.x = c(21, "plain", "black"),

font.y = c(21, "plain", "black"),

font.tickslab = c(21, "plain", "black"),

tables.height = 0.3,

risk.table.y.text = TRUE,

risk.table.fontsize = 6,

tables.theme = theme(axis.text = element_text(size = 21))

)

PSM_ggsurv_III_4

pdf("MIMIC_III_Quantile4.PDF")

PSM_ggsurv_III_4

dev.off()

**Reference**

Evans, L., Rhodes, A., Alhazzani, W., Antonelli, M., Coopersmith, C.M., French, C., Machado, F.R., Mcintyre, L., Ostermann, M., Prescott, H.C., Schorr, C., Simpson, S., Wiersinga, W.J., Alshamsi, F., Angus, D.C., Arabi, Y., Azevedo, L., Beale, R., Beilman, G., Belley-Cote, E., Burry, L., Cecconi, M., Centofanti, J., Coz Yataco, A., De Waele, J., Dellinger, R.P., Doi, K., Du, B., Estenssoro, E., Ferrer, R., Gomersall, C., Hodgson, C., Møller, M.H., Iwashyna, T., Jacob, S., Kleinpell, R., Klompas, M., Koh, Y., Kumar, A., Kwizera, A., Lobo, S., Masur, H., Mcgloughlin, S., Mehta, S., Mehta, Y., Mer, M., Nunnally, M., Oczkowski, S., Osborn, T., Papathanassoglou, E., Perner, A., Puskarich, M., Roberts, J., Schweickert, W., Seckel, M., Sevransky, J., Sprung, C.L., Welte, T., Zimmerman, J., and Levy, M. (2021). Surviving sepsis campaign: international guidelines for management of sepsis and septic shock 2021. *Intensive Care Med* 47**,** 1181-1247.

Hu, W., Chen, H., Ma, C., Sun, Q., Yang, M., Wang, H., Peng, Q., Wang, J., Zhang, C., Huang, W., Xie, J., and Huang, Y. (2023). Identification of indications for albumin administration in septic patients with liver cirrhosis. *Crit Care* 27**,** 300.

Reyna, M.A., Josef, C.S., Jeter, R., Shashikumar, S.P., Westover, M.B., Nemati, S., Clifford, G.D., and Sharma, A. (2020). Early Prediction of Sepsis From Clinical Data: The PhysioNet/Computing in Cardiology Challenge 2019. *Crit Care Med* 48**,** 210-217.

Seymour, C.W., Liu, V.X., Iwashyna, T.J., Brunkhorst, F.M., Rea, T.D., Scherag, A., Rubenfeld, G., Kahn, J.M., Shankar-Hari, M., Singer, M., Deutschman, C.S., Escobar, G.J., and Angus, D.C. (2016). Assessment of Clinical Criteria for Sepsis: For the Third International Consensus Definitions for Sepsis and Septic Shock (Sepsis-3). *Jama* 315**,** 762-774.

Yang, M., Liu, C., Wang, X., Li, Y., Gao, H., Liu, X., and Li, J. (2020). An Explainable Artificial Intelligence Predictor for Early Detection of Sepsis. *Crit Care Med* 48**,** e1091-e1096.

Zhang, Z., Ho, K.M., and Hong, Y. (2019). Machine learning for the prediction of volume responsiveness in patients with oliguric acute kidney injury in critical care. *Crit Care* 23**,** 112.
